# Microfluidic techniques for isolation, formation, and characterization of circulating tumor cells and clusters

**DOI:** 10.1063/5.0093806

**Published:** 2022-07-15

**Authors:** Celine Macaraniag, Qiyue Luan, Jian Zhou, Ian Papautsky

**Affiliations:** Department of Biomedical Engineering, University of Illinois Chicago, Chicago, Illinois 60607, USA

## Abstract

Circulating tumor cell (CTC) clusters that are shed from the primary tumor into the bloodstream are associated with a poor prognosis, elevated metastatic potential, higher proliferation rate, and distinct molecular features compared to single CTCs. Studying CTC clusters may give us information on the differences in the genetic profiles, somatic mutations, and epigenetic changes in circulating cells compared to the primary tumor and metastatic sites. Microfluidic systems offer the means of studying CTC clusters through the ability to efficiently isolate these rare cells from the whole blood of patients in a liquid biopsy. Microfluidics can also be used to develop *in vitro* models of CTC clusters and make possible their characterization and analysis. Ultimately, microfluidic systems can offer the means to gather insight on the complexities of the metastatic process, the biology of cancer, and the potential for developing novel or personalized therapies. In this review, we aim to discuss the advantages and challenges of the existing microfluidic systems for working with CTC clusters. We hope that an improved understanding of the role microfluidics can play in isolation, formation, and characterization of CTC clusters, which can lead to increased sophistication of microfluidic platforms in cancer research.

## I. INTRODUCTION

Circulating tumor cells (CTCs) and their clusters disseminate to distant sites in the body through the bloodstream as part of the metastatic process. Their occurrence is extremely rare, often <100 CTCs or <10 clusters per 10 × 10^6^ leukocytes and 5 × 10^9^ erythrocytes in 1 ml whole blood.^1–5^ Nevertheless, their occurrence far exceeds the number of actual metastatic lesions in patients, supporting the notion that very few CTCs overcome the harsh vascular environment and successfully metastasize onto a secondary site.^2^ Clinical studies have indicated that CTC clusters have prognostic value associated with predicting treatment resistance and survival outcomes,^6–8^ suggesting their importance in developing personalized and novel cancer therapies. These cells can offer information on differences in the genetic profiles, somatic mutations, and epigenetic changes compared to cells in primary tumor and metastatic sites.^9,10^ Yet, detection of CTC clusters has been associated with worse clinical outcomes and higher (estimated as 20–50 fold greater) metastatic potential.^2^ The mechanisms that lead CTCs to acquire an invasive phenotype are yet to be fully understood, and due to the inherent rarity of CTCs and CTC clusters, extensive characterization is limited.

**FIG. 1.**
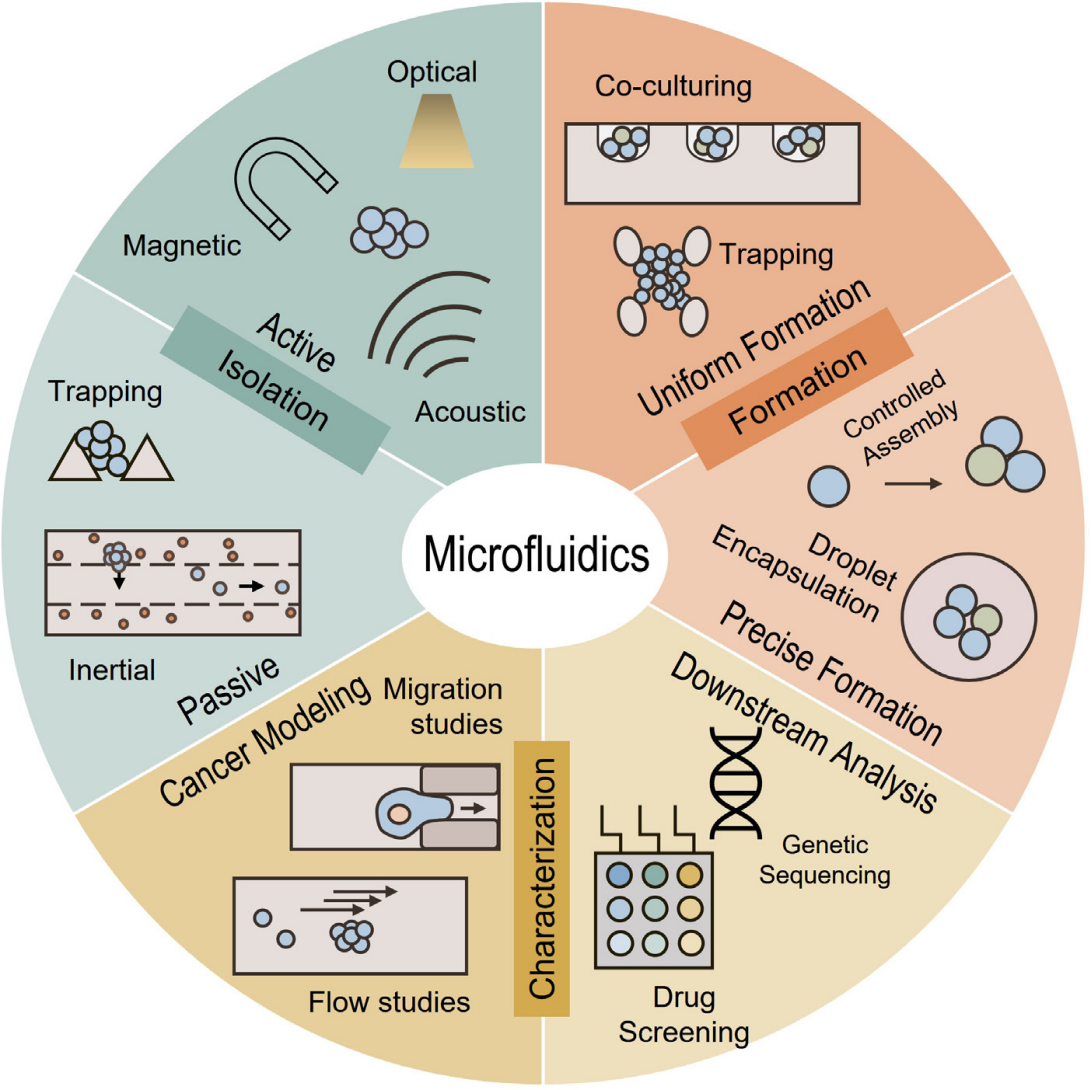
Microfluidic isolation, formation, and characterization of CTC clusters for cellular and molecular investigations of cancer metastasis or disease monitoring.

CTCs and clusters are typically isolated directly from blood using immunoselection or flow cytometry, but microfluidic isolation is an attractive alternative only requiring smaller volumes of samples and less procedural steps suitable for diagnosis, molecular analysis, and *in vitro* studies. Microfluidic systems have proven capability in isolation, formation, and characterization of individual cells,^11^ spheroids,^12–14^ and organoids^15–17^ over the past few years (Fig. 1). Microfluidic methods of isolation and analysis have become more diverse with both physical and biomarker-based techniques to capture, label, and phenotype CTCs. These techniques include the physical and biomarker capture of primary CTCs,^18^ induction of physical parameters to assess phenotype,^19^ deformability assays,^20^ protein quantification,^21^ drug response,^22^ and many others.

In addition, *in vitro* modeling of metastasis is possible by fine spatiotemporal control over a cellular microenvironment. Microfluidics has also been used to study the effects of flow and shear stress on CTCs with the use of modified channel geometries, flow rates, and reagent compositions. With limited sample quantities, further characterization may require the *in vitro* formation of clusters from either expanded patient-derived cells or cancer cell lines integrated with downstream processes such as drug screening, molecular profiling, and/or cancer modeling. Differences between primary and cell line-formed clusters may also need more investigation; therefore, conducting both methods may be done in complementary with each other for more physiologically accurate modeling of CTC clusters. A combination of *in vitro* metastasis assays, *in vivo* preclinical models, and analysis of patient-derived cells could potentially elucidate the process of metastasis and lead to more effective cancer therapies.

In this review, we will highlight the microfluidic techniques in isolating, forming, and characterizing CTC clusters from patient-derived samples, preclinical *in vivo* models, and cancer cell lines. Others have already covered the microfluidic strategies for *in vitro* organoids and cell spheroid formations for tumor modeling or drug screening applications.^12–14^ However, these discussions revolve around modeling solid tumors and their microenvironment using miniature tissue constructs. While increased attention has been paid to the topic of CTC clusters, few reviews specifically focus on CTC clusters. Indeed, some reviews discuss cell isolation and enrichment techniques^9,23,24^ while others focus exclusively on drug screening in solid and liquid biopsies.^25^ In this review, our goal is to highlight the contributions of microfluidics in all three aspects: isolation, formation, and characterization of CTC clusters. We will first provide a brief discussion of some studies around the biology of CTC clusters and then illustrate the role microfluidics can play in elucidating metastatic mechanisms in the hope of developing improved therapies. Finally, we will discuss the current challenges and future perspectives of microfluidic technologies for cancer and metastasis research.

## II. CTC CLUSTERS FOR CANCER RESEARCH

CTCs can offer much real time information on the status of cancer in a patient. These cells have been used in *in vivo* models of metastasis to further elucidate the process and aid in developing treatments. Studies have provided evidence that CTC clusters detach as multicellular aggregates from the primary tumor and hold necessary information on the pathways of metastasis.^2^ It is, thus, of importance to identify the phenotypic traits and molecular features of CTCs and CTC clusters that facilitate metastasis seeding and the basis of the interactions of CTCs with other cell types such as immune and stromal cells.

In addition to CTC clusters, other cellular aggregates used for modeling of solid tumors include organoids and spheroids. Organoids are 3D self-organizing tissue constructs, usually embedded in a 3D matrix, derived from healthy and tumor tissue samples,^26^ CTCs,^27^ or engineered with embryonic or induced pluripotent stem cells.^28,29^ Organoids also offer flexibility, as they are compatible with high-throughput analyses and are amenable to genetic modification.^24^ Similarly, spheroids are aggregates of cells with a uniform geometry that mimics small tissue lesions but are less complex than organoids.^30^ There are fundamental differences in the structure and microenvironment between most cell spheroids and CTC clusters (Table I). Tumor spheroids exhibit steep gradients, cell-matrix attachments, and a necrotic core mimicking tumor areas away from blood vessels, which may not necessarily reflect the properties and behavior of CTC clusters.^11,16^ Therefore, careful design must be considered to better represent the characteristics of intravascular cell clusters that are exposed to other blood cells and fluidic stresses during their transit in circulation.^31^

**TABLE I.** Comparison of cellular formations in cancer research.

		Cell aggregates		
	Single cells	Clusters	Spheroids	Organoids
	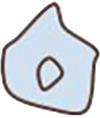	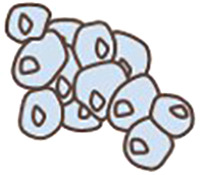	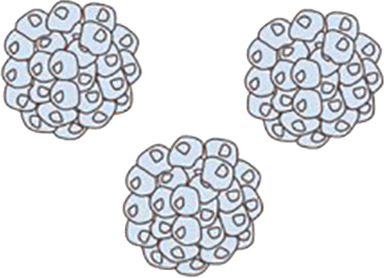	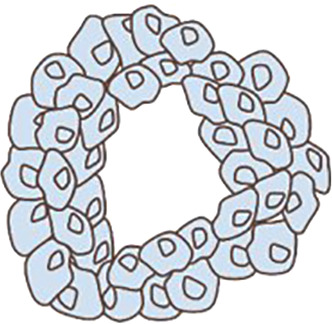
Characteristics	Single cells may be isolated by physical-based or affinity methods Useful in single-cell resolution next-generation sequencing Amenable for high-throughput technologies in imaging, mass spectrometry and DNA- and RNA-sequencing	Aggregates of cancer cells that depart from solid tumors and enter the bloodstream.^86^^^Isolated from whole blood or formed *in vitro.*CTC clusters display distinct gene expression profiles and dissemination modes compared to single CTCs and play a role in cancer progression.^86^	3D spherical cell aggregates that model *in vivo* solid tumors. Larger spheroids have central necrosis and regions of hypoxia.^12^^^Can be formed *in vitro* by 3D culture, hanging drop, microwell, droplet Mimic tissues with cell–cell interactions as well as diffusional limits to mass transport of drugs, nutrients, and other factors.^12^	3D self-organizing cellular constructs with tissue structure and function. Derived from adult organs or embryonic and adult stem cells.^134^^^Demonstrate functionality such as muscle contractility, epithelial barrier function, neuronal activity, hepatocyte detoxification, gastric acid secretion, and secretion of insulin by beta cells.^134^
Related microfluidic reviews	Pei *et al.*^11^ and Sharma *et al.*^135^	Sharma *et al.*^135^	Mehta *et al.*,^12^ Moshkayan *et al.*,^13^ and Vadivelu *et al.*^14^	Ayuso *et al.*,^136^ Duzagac *et al.*,^137^ Kim *et al.*,^138^ and Velasco *et al.*^139^

The main interest in studying CTC clusters and their importance includes the understanding of interactions between CTCs and blood cells,^32^ identifying the characteristics that CTCs attain during their transit in the bloodstream,^33^ the prognostic value of CTC cluster analysis for personalized medicine,^6^ and the efficacy of anti-cancer drugs in targeting CTC clusters.^25^ Although much has yet to be learned on the specific mechanisms of the metastatic process, some studies have slowly begun to reveal some insights. Preclinical mouse models have been vital in many of these mechanistic studies of metastasis;^34–36^ therefore, isolation of CTCs from them is routinely done in the research setting. Studies reveal that CTC clusters can exhibit partial epithelial-to-mesenchymal transition (EMT), which may be associated with the suppression of anoikis since they are able to retain their intercellular junctions, and therefore, maintain physical stability. EMT switch of CTCs may also be modulated by survival and drug resistance pathways initiated by treatment.^37^ The presence of other leukocytes in the bloodstream could even serve as a layer of protection during their transit.^32^ Contact between platelets and CTCs is sufficient to induce metastatic gene expression signature, an EMT-like transformation, and invasive behavior.^38^

Tumors inherently exhibit significant genetic and transcriptomic heterogeneity as well as in epigenetics where inherent stochasticity of transcription may affect gene expression.^39,40^ In comparing the transcriptome profiles of neutrophil-associated CTCs against those of CTCs alone, Szczerba *et al.*^34^ observed differentially expressed genes in CTC-neutrophil clusters that displayed enrichment in positive regulators of cell cycle and DNA replication compared to lone CTCs, which lead to the efficient formation of metastasis.^34^ These findings motivate the need for even more mechanistic studies to fully uncover the metastatic process. Many *in vitro* studies of CTCs and metastasis have benefited from microfluidics through isolation, culture, and analysis systems.

Challenge arises in the preservation of viability for *ex vivo* CTC analysis. *In vivo* studies have shown that it only takes a few viable cells to survive circulation for metastasis to occur.^41,42^ In patient samples, CTC numbers and viability can vary as well (<10–1500 CTCs breast^43^ and non-small cell lung cancer patients^43,44^). Metastatic propensity can be influenced by the physical characteristics that single CTCs and CTC clusters possess, and metastasis may favor the survival and growth of a few subpopulations of these cells.^41^ Cancer cells may also exploit and/or be resistant to the physical forces present in the circulation to successfully seed in distant metastases.^45,46^ For example, flow rates, vessel size, circulating time, and shear stress can influence the survival of CTCs and control seeding patterns.^47,48^ These factors, therefore, might be of importance when dealing with CTCs *in vitro* and/or *ex vivo.* In fact, some have started to study these influences using microfluidic methods to induce such physical parameters.^19,45,48,49^

Another important aspect to consider is reliable methods of assessment of viability. In *ex vivo* capture, only a small fraction of captured CTCs will be viable for cell analysis and cell expansion.^41^ This necessitates for high rates of cell viability in microfluidic devices. In *ex vivo* studies, the cell release and transfer of samples and reagents can contribute to the death of a few cells. Indeed, for microfluidic capture systems, previous works have shown over 97% cell viability is achieved in microfluidic platforms.^50^ In triangular traps, the release of clusters under 250 ml/h reverse flow had no negative effect on cell viability.^51^ In another example, LM2 cells had achieved up to >97% viability in a microwell valve system.^21^

**FIG. 2.**
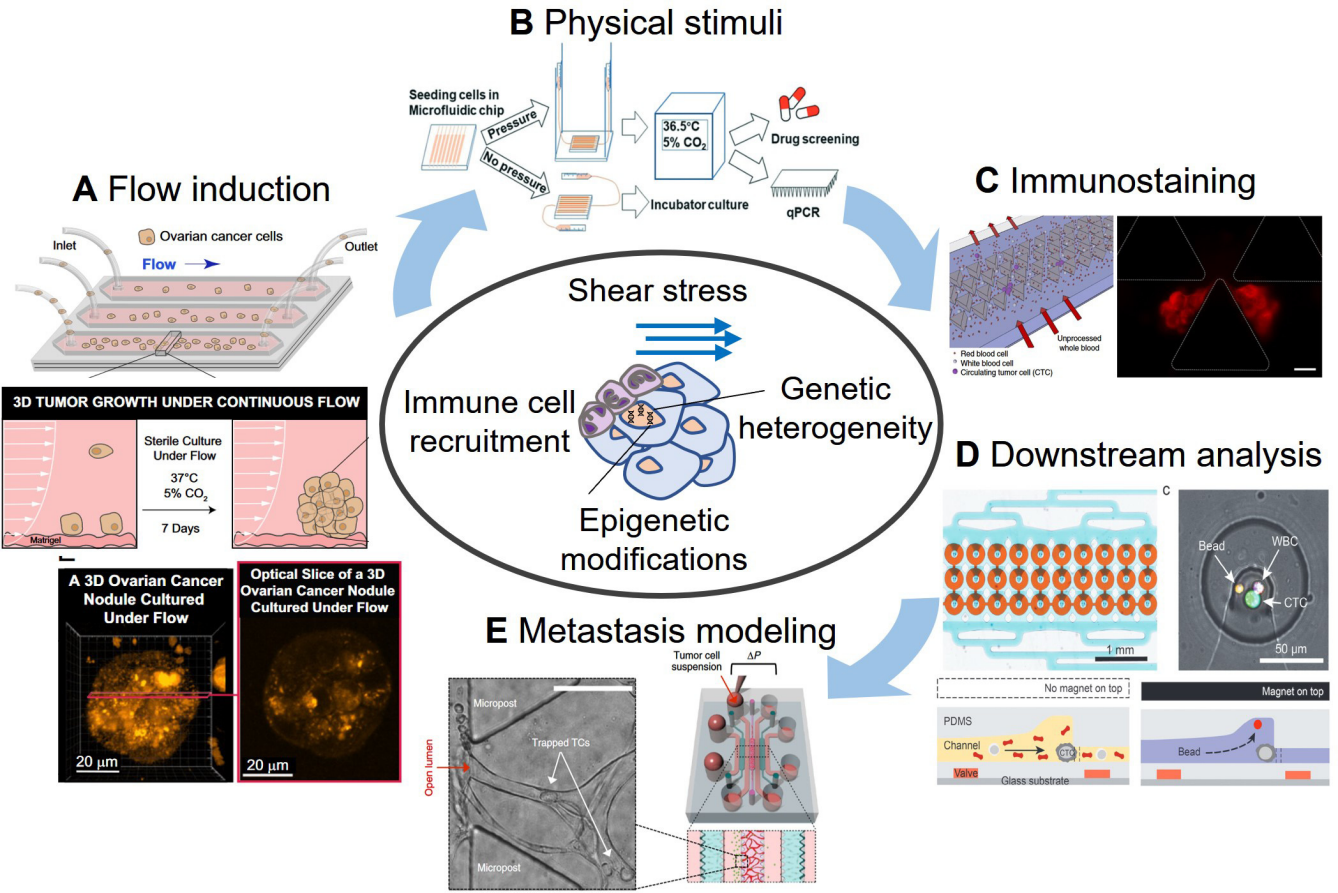
Microfluidic techniques to evaluate relevant factors affecting CTC clusters. (a) Flow experiments in microfluidic chip to study the effect of sustained flow on the growth and molecular features of 3D ovarian cancer nodules. Reproduced with permission from Rizvi *et al.*, Proc. Natl. Acad. Sci. **110**, 22 (2013). Copyright 2013 Authors, licensed under a Creative Commons Attribution (CC BY) license.^19^ (b) Study of hydrostatic pressure effects on human breast cancer cell drug resistance. Republished with permission from Shang *et al.*, Lab Chip **21**, 4 (2021). Copyright 2021 Royal Society of Chemistry and Copyright Clearance Center, Inc.^111^ (c) On-chip immunostaining of CTC clusters after capture. Reprinted by Sarioglu *et al.*, Nat. Methods **12**, 7 (2015). Copyright 2015 Springer Nature Customer Service Center GmbH.^51^ (d) Microfluidic protein quantification using a bead-based immunoassays. Reproduced with permission from Armbrecht *et al.*, Adv. Sci. **7**, 11 (2020). Copyright 2020 Authors licensed under a Creative Commons Attribution (CC BY) license.^21^ (e) *In vitro* model of microcirculation to study cell arrest, transendothelial migration and early micrometastases formation. Reprinted with permission from Chen *et al.*, Nat. Protoc. **12**, 5 (2017). Copyright 2017 Springer Nature Customer Service Center GmbH.^140^

Microfluidic systems have also made evaluation of CTCs and CTC clusters achievable in a high-throughput manner with the ability to recapitulate the cancer microenvironment (Fig. 2). Ideally, the integration of CTC analyses with isolation and cell culture could make these microfluidic systems functional in the clinical setting. Below, we summarize the microfluidic techniques used in obtaining and analyzing CTC clusters for cancer research.

## III. ISOLATION OF CTC CLUSTERS WITH MICROFLUIDIC SYSTEMS

Microfluidic isolation technologies make it possible to extract primary cell specimens from patient blood that can be used for biological investigations and disease diagnosis and/or prognosis. In addition, these systems may be integrated with cell culture systems to expand patient-derived cells and develop CTC cell lines for prolonged *ex vivo* studies such as cell invasiveness and metastatic competency.^52^ The two main methods used to obtain primary cells are the solid and liquid biopsy. Solid biopsy is the traditional sampling method, where a small amount of tissue is extracted from the body to obtain patient-derived primary cells. Post-analysis of these tissues requires their dissociation into single cells, which may be done chemically, mechanically, or through microfluidics.^53^ However, solid biopsy is an invasive technique that cannot be repeated frequently and can present challenges depending on the anatomical structure of the tumor or its location. Therefore, much attention has been paid to liquid biopsy, which involves extraction of bodily fluids, such as blood or pleural effusion, for analysis of biomarkers, including CTCs, circulating tumor DNA (ctDNA), and extracellular vesicles (EVs).^54^

Liquid biopsy is gaining popularity due to its minimally invasive nature, which allows for routine disease monitoring over time and more frequently. Microfluidic techniques are especially advantageous in liquid biopsy as they only require small sample volumes and minimal manual pipetting. In addition, they can be integrated for further downstream molecular analysis, *in vitro* studies, or tracking of tumor response to treatment. Microfluidic methods of isolating CTCs and CTC clusters include sized-based,^55,56^ marker-dependent,^18,57^ and active techniques.^60^ These are summarized in Table II.

**TABLE II.** Methods for microfluidic isolation of CTCs and clusters.

	Principle	Efficiency	Purity	Throughput	Advantages	Disadvantages
Sized-based trapping	Hydrodynamic capture through microwells^92,93^ and micropost trapping^51^	∼40–99% (Increased efficiency with increasing cluster size)	⋯	200 ml/min, −2.5 ml/h	High-efficiency, label-free	Low-throughput, low purity, and low specificity
Sized-based flow separation	Deterministic lateral displacement (DLD),^4^ shear-induced diffusion (SID),^50,72^ and inertial focusing^3,67–69,73^	∼37%–99%	57%–94%	0.2–0.5 ml/min	High-throughput, high-efficiency, label-free	Low specificity, low-mid purity
Marker-dependent	Antibody^1,58,75,76,141^ and nanoparticle^59^ coated surfaces	∼60%–98%	∼14%–86%	1–2.5 ml/h	High specificity	Low throughput, low purity, low efficiency, difficult to retrieve cells
Active	Capture through optical,^83^ magnetic,^84^ acoustic,^78,79,82^, and dielectrophoretic^83,85^	∼71%–90%	84%–91.5%	1.2–30 ml/h	High purity, minimized shear force, label-free	High efficiency, low throughput, complex setup

Some techniques involve integration of cell sorting with trapping,^50^ where after sorting, cells or clusters may be trapped using microwell, micropost, microfiltration, or microchamber methods without external force.^61^ Isolation solely by trapping is also an effective method of CTC capture.^61^ For example, Hosokawa *et al.*^62^ used a microcavity array system to capture single and clustered CTCs.^62^ Sarioglu *et al.*^51^ developed the cluster chip, a micropillar device made of bifurcating traps specifically to trap cell clusters from unprocessed blood.^51^ These capture methods immobilize single cells and clusters in a controllable and independent manner enabling single-cell analysis.^61^ Microfiltration has also been used in CTC capture, which creates less mechanical stress on the CTCs.^62–64^ Zhou *et al.*^64^ achieved a high capture efficiency of 78%–83% and a cell viability of 71%–74% in their separable bilayer microfiltration device.

**FIG. 3.**
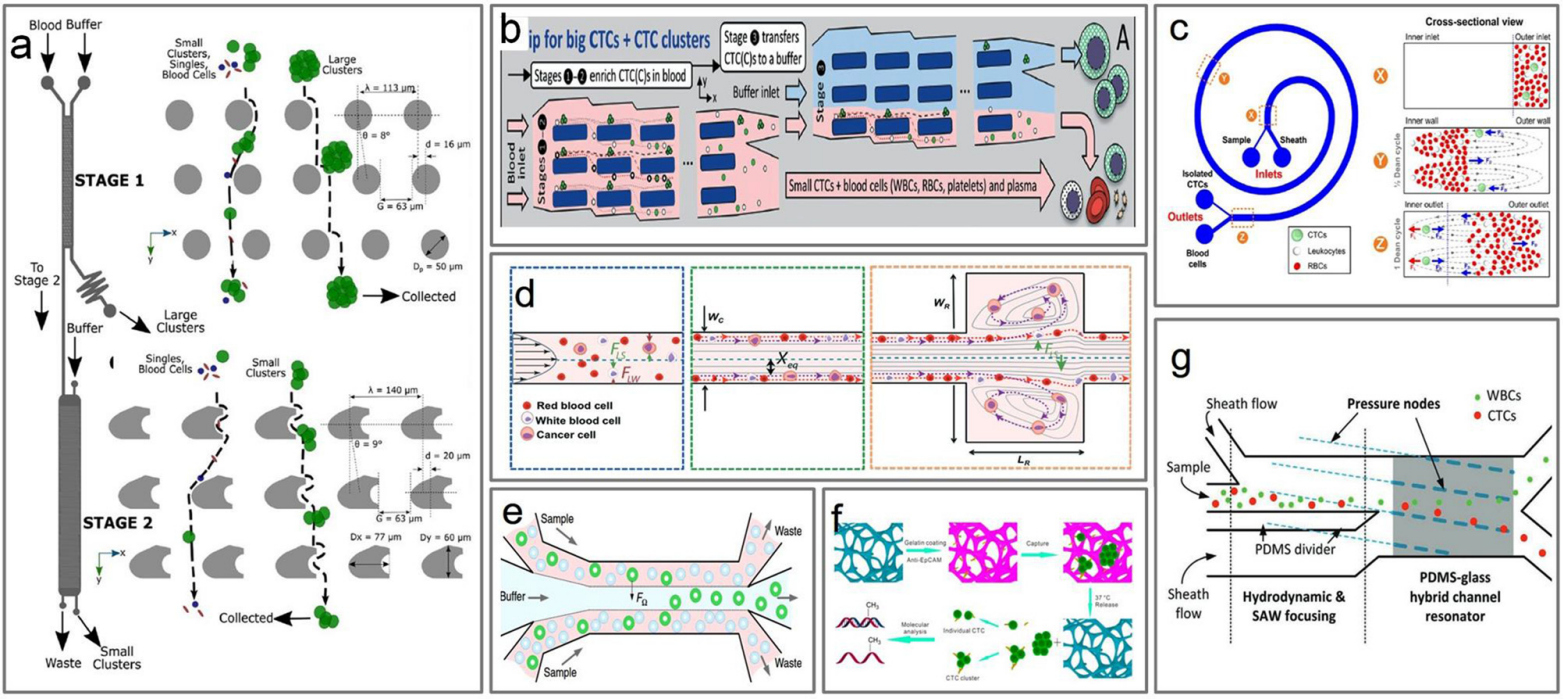
Microfluidic capture of single and clustered CTCs. (a) Two-stage cluster capture array of cylindrical micropillars. Stage 1 deflects large clusters while stage 2 deflects small clusters from other blood cells using deterministic lateral displacement. Reproduced with permission from Au *et al.*, Sci. Rep. **7**, 1 (2017). Copyright 2017 Authors, licensed under a Creative Commons Attribution (CC BY) license.^4^ (b) Size-based CTC and cluster sorter built from enlarged non-equilibrium inertial separation arrays. Republished with permission from Edd *et al.*, Lab Chip **20**, 3 (2020). Copyright 2020 Royal Society of Chemistry and Clearance Center, Inc.^67^ (c) CTC isolation using the spiral inertial microfluidic device where smaller cells migrate toward the inner wall, then back to outer wall again, while the larger CTCs experience additional strong inertial lift forces and focus along the microchannel inner wall. Reproduced with permission from Hou *et al.*, Sci. Rep. **3**, 1 (2013). Copyright 2013 Authors, licensed under a Creative Commons Attribution (CC BY) license.^69^ (d) In the Vortex chip, particles migrate to lateral equilibrium positions depending on the channel cross section, where the wall effect is reduced in the reservoir. Republished with permission from Sollier *et al.*, Lab Chip **14**, 1 (2014). Copyright 2014 Royal Society of Chemistry Clearance Center, Inc.^71^ (e) Inertial separation of CTCs and clusters, where they are focused to the center of a straight channel. Reproduced with permission from Zhou *et al.*, Microsyst. Nanoeng. **5**, 1 (2019). Copyright 2019 Authors, licensed under a Creative Commons Attribution (CC BY) license.^73^ (f) Capture and release of CTC cluster in a 3D scaffold chip. Reproduced with permission from Cheng *et al.*, Anal. Chem. **89**, 7924–7932 (2017).^76^ Copyright 2017 American Chemical Society. (g) Acoustic separation of CTCs from WBCs. The cells are separated due to difference in the lateral shift. Republished with permission from Wu *et al.*, Small **14**, 32 (2018). Copyright 2018 John Wiley and Sons, Clearance Center, Inc.^82^

Other isolation methods are sized-based techniques^55,56^ that avoid complications related to biomarker inconsistency since detection is not based on specific surface markers but rather use other physical properties such as size or deformability to isolate target cells.^65^ One label-free method is the deterministic lateral displacement (DLD),^66^ a passive technique that separates particles based on size. Au *et al.*^4^ used the DLD method in a two-stage device with the first stage separating large clusters and the second stage for smaller clusters that were initially deflected by the first stage using asymmetric pillars [Fig. 3(a)].^4^ This technique preserved the integrity of clusters while also minimizing damage that may lead to processing bias. Edd *et al.*^67^ developed a non-equilibrium inertial separation array that combines inertial focusing with repetitive flow-shifting isolating CTC clusters from large volumes of minimally diluted whole blood [Fig. 3(b)].^67^ The device isolated spiked CTC clusters from >30 ml/h of whole blood with 80% efficiency and an on-chip yield of ∼100%.^67^ However, the main disadvantage of the size-based separation methods is the loss of smaller cells, which can result in loss of valuable information from the patient.^68^

Centrifugal forces with Dean flow also enable the continuous focusing of larger cells.^55,56^ Hou *et al.*^69^ used a spiral channel isolation device exploiting Dean migration and inertial separation in curvilinear channels, achieving >85% separation efficiency [Fig. 3(c)]. Capture of intact clusters of MCF-7 breast cancer cells was achieved even with high flow conditions due to the short transit time within the channel, which prevented the breakup of clusters.^69^ In another method developed by the Di Carlo group,^70^ microscale vortices and inertial focusing were employed to passively isolate and concentrate larger cells [Fig. 3(d)].^71^ They optimized this method for high-purity extraction by systematically varied parameters, including channel dimensions and flow rates, to arrive at an optimal device for maximum trapping efficiency and purity (57%–94% purity).^71^ Kulasinghe *et al.*^3^ employed inertial migration using a simple straight channel device to isolate head and neck CTCs' clusters from whole blood followed by DNA fluorescence *in situ* hybridization (FISH) [Fig. 3(e)].^3^ As another method of passive separation, Hayashi *et al.*^63^ used shear-induced diffusion (SID) of cells from concentrated suspensions.^62^ This technique was used to separate CTCs from unprocessed whole blood in a straight, rectangular microfluidic channel integrated with a cell trapping and culture chamber. The device integration effectively streamlines cell separation, capture, staining, or *in situ* culture with little manual interference.^63^

Marker-dependent techniques for cell isolation^18^ make use of epithelial cell adhesion molecule (EpCAM) expressed on the surface of tumor cells.^1,74^ This technique is also applied in the first CTC isolation system, CellSearch, to be approved by the U.S. Food and Drug Administration (FDA). Anti-EpCAM magnetic particles conjugate to tumor cells and be immobilized by a magnetic force.^1^ In microfluidic devices, surface functionalization with antibodies allows for marker-dependent capture of CTCs and CTC clusters. Stott *et al.*^58^ developed the herringbone (HB)-chip with surface-lined anti-EpCAM antibodies that captured CTCs and CTC clusters with 79% ± 4.5% efficiency at 0.12 ml/h flow rate.^58^ The herringbone design induces microvortices that disrupt the laminar flow streamlines and, therefore, increase the number of cell interactions with the antibody-coated surfaces. Jiang *et al.*^75^ then used the HB-chip in a two-step process for platelet covered CTC-leukocyte cluster isolation based on immunoaffinity to platelets.^75^ Microfluidic devices can also be scaffolded for a 3D capture of CTC clusters.^57,76^ For example, Cheng *et al.*^76^ achieved >80% capture efficiency and 60%–70% recovery ratio in spiked samples, followed by DNA and RNA methylation analysis of the cells [Fig. 3(f)]. To perform downstream analysis right after capture, it is then necessary to release cells from the surface. A ligand-exchange reaction was used by Park *et al.*^59^ using a modified the HB-chip with gold nanoparticle coating to allow for easy detachment of the captured CTCs. The metal−thiol interactions can be disrupted in the presence of excess thiol molecules that resulted in the release of cells.^59^ Though these marker-dependent techniques can be highly specific, a limitation to this approach is the reduced efficiency due to downregulation EpCAM in some CTCs. Therefore, alternative methods that do not require the EpCAM expression may be necessary to isolate some CTCs and CTC clusters.^77^

So far, we have focused on passive separation methods, yet active methods can too be used in a label-free format and can maintain the integrity of cells and clusters.^78,79^ Active methods^60^ use external force such as acoustic, magnetic, optical, or dielectrophoretic to separate and isolate cell clusters from blood or buffer. The acoustic-based method uses standing surface acoustic waves to gently separate CTCs from white blood cells (WBCs), preserving the phenotype and genotype of the cell.^80,81^ Wu *et al.*^82^ successfully applied this technique to separate CTCs and CTC clusters from blood samples collected from patients with metastatic prostate cancer.^82^ Chiu *et al.*^83^ used optically induced dielectrophoresis (ODEP) to separate CTC clusters [Fig. 3(g)].^83^ Their device used alternating current voltage between the top and bottom surfaces of the ODEP system and generated a non-uniform electric field when illuminated. The electric field interacts and manipulates an electrically polarized microparticle where >80% purity is achieved.^83^ Lin *et al.*^84^ applied lateral and vertical magnetic force to separate white blood cells from whole blood at a flow rate of 20 *μ*l min^−1^ and achieved the final separation purity 93% ± 1.7% and viability up to 97.5% ± 1.8%. Do *et al.*^85^ developed a microfluidic DEP using a gold electrode structure, which is used to concentrate cells suspended in the chamber by DEP and a stepping electric field. The target cells are captured by an electrode immobilized by anti-EGFR, which has high affinity toward the target cells. In general, active methods are usually high in efficiency; however, compared to passive methods, some major drawbacks to these systems are that they are more complex in operation, usually lower in throughput, and require pretreatment of blood.

Microfluidic isolation systems have progressed in recent years and have demonstrated efficient separation of target cells. In addition, many improvements have been made in applying these systems to the isolation of extremely rare cells from biological samples, proving their value in the clinical and laboratory setting. As discussed here, there are advantages and disadvantages to each method of isolation, and therefore, careful decision must be made for specific applications. However, even with advancements in these methods, due to the rarity of CTC clusters, the sole isolation of these cells is not sufficient for extensive research and/or high-throughput screening. In Sec. IV, we discuss potential ways to form and expand such cell clusters to extend their use in the laboratory.

## IV. FORMATION OF CTC CLUSTERS WITH MICROFLUIDICS

The potential clinical significance of CTC clusters motivates the need for accurate *in vitro* cancer models to survey how CTC clusters survive and how to target them effectively with anti-metastatic treatments. In general, CTC clusters can either be homotypic or heterotypic, where heterotypic clusters can include other immune and stromal cells. Heterotypic clusters are much rarer compared to single CTCs and homotypic CTC clusters.^86^ Although rare, studies suggest that heterotypic clusters may be significant in initiating metastasis.^34,86^ Therefore, it is imperative to explore the potential of these clusters as clinical tools for research. Simple microfluidic systems have been proven effective in the culture of single cells^87^ and *in vitro* formation of cell spheroids.^88^ Other methods of aggregate formations have been performed using hanging drop methods,^88^ liquid marbles,^89^ non-adherent plates,^30,51^ and droplet formation^90^ techniques; however, microfluidic techniques offer much more uniform and precise formation of CTC clusters.

Forming *in vitro* CTC clusters with readily available cancer cell lines can also be used for both investigation of cell cluster behavior and drug screening where cell response can be assessed in a very high-throughput manner. For example, cancer cell lines, such as NCI-H187 (small cell lung cancer) and NCI-H2122 (non-small cell lung cancer), exhibit similar features as primary CTCs and, therefore, can be alternatives for patient-derived CTCs.^31^ Although these may not completely replace primary cells, they can be used for optimizing *in vitro* models as well as complementing preclinical mouse studies and clinical sample analysis. Moreover, microfluidic cell sorting and capture devices can conveniently be integrated with on-chip cell culture systems^50^, to ease the processing of samples. Perhaps the simplest approach in forming clusters would be the use of microwells, which relies on the sedimentation of cells into individual compartments allowing for fine spatial control. Spheroid and organoid formations are commonly formed with microwell techniques.^15,16,22^ Jung *et al.*^15^ established lung cancer cell organoids derived from small-cell lung cancer (SCLC) patients in a microwell system that is able to load, expand, and identify drug responses under physiologically relevant flow conditions.^15^ Dadgar *et al.*^16^,^22^ demonstrated the formation of spheroids with a relatively low seeding density, demonstrating the utility of the microfluidics to be used with the limited cell numbers. Microwells also allow for high-throughput single-cell analysis due to the ease in the fabrication of many individual compartments in one chip. Size and cross-sectional profiles of microwells can be easily tuned to meet the requirements for specific cell culture applications such as the formation of CTC clusters.^22^

**FIG. 4.**
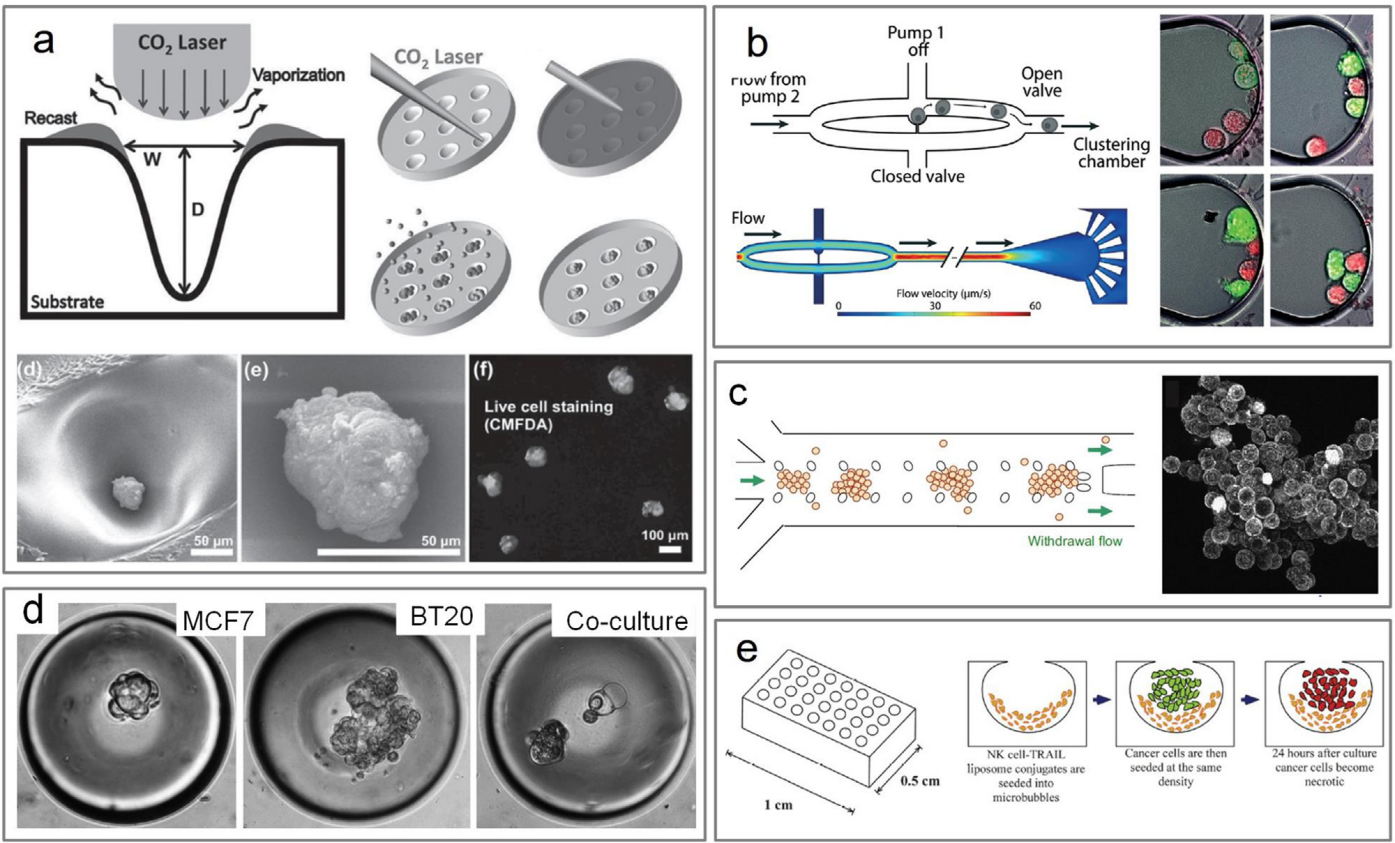
*In vitro* formation of clusters in microfluidic devices. (a) Laser ablation fabrication process of concave microwells for the formation of 3D aggregates. Republished with permission from Tu *et al.*, Adv. Healthcare Mater. **3**, 4 (2013). Copyright 2013 John Wiley and Sons, Clearance Center, Inc.^91^ (b) Ejection of cells from single-cell traps where lock arrows denote the flow paths of cells dislodged from the trap flowing toward the clustering chamber. Republished with permission from Fatsis-Kavalopoulos *et al.*, Lab Chip **19**, 6 (2019). Copyright Royal Society of Chemistry Clearance Center, Inc.^95^ (c) Schematic illustration of *in situ* formation and immobilization of 3D multi-cellular aggregates in a microchannel. Reprinted with permission from Ong *et al.*, Biomaterials **29**, 3237–3244 (2008). Copyright 2008 Elsevier.^100^ (d) Bright field images of CTC clusters grown in PDMS microbubbles. Reproduced with permission from King *et al.*, Am. J. Physiol.-Cell Physiol. **308**, 10 (2015). Copyright 2015 Authors, licensed under a Creative Commons Attribution (CC BY) license.^101^ (e) Cell culture microbubbles formed in PDMS for lymph node micrometastases model. Republished with permission from Chandrasekaran *et al.*, Lab Chip **14**, 1 (2014). Copyright 2014 Royal Society of Chemistry Clearance Center, Inc.^102^

Parameters that contribute to the proper formation of cell clusters in microwells include cell seeding density, microwell dimensions, and device surface properties. Tu *et al.*^91^ used CO_2_ laser ablation on polystyrene substrates to create concave microwells for the formation of A549 lung cancer cell clusters that are about 50 *μ*m in size [Fig. 4(a)]. In their study, A549 cell aggregates were successfully generated in polystyrene microwells, where aggregate size was controllable and able to form about ∼40–80 *μ*m-sized aggregates.^91^ It has also been demonstrated to form cell clusters directly after extraction from patient blood through red blood cell (RBC) lysis. Short term culture of clusters after RBC lysis from multiple cancer types was achieved by Balakrishnan *et al.*^92^ using ellipsoidal agar microwells with cultures maintained for 3 weeks. They assessed the expressions of cytokertatins (CK) in clusters that vary in compactness and found that tighter clusters showed greater therapy resistance.^92^ Khoo *et al.*^93^ created a microwell system capable of co-culturing CTCs with the white blood cells from the same patient.^93^ The device consisted of three layers, including a concentration gradient generator, a barrier layer that separates each column of inverted dome-shaped elliptical microwells into an individual channel, and a layer of microwells that contain the CTC clusters. This technology can derive CTC clusters from liquid biopsies without prior enrichment of the CTCs.^93^

For the precise formation of heterotypic clusters, more complex systems involving valving, trapping, and/or droplet formation techniques are used. Microvalves can facilitate controlled cell culture and drug assays such as the work of Desyatnik *et al.*,^94^ where pneumatic microvalves were combined with cell culturing of MCF-7 and 293T cells and drug microarray [Fig. 4(b)]. The cells were allowed to aggregate and then cultured for 24 h where they found substantial cell clustering.^94^ Fatsis-Kavalopoulos *et al.*^95^ created a hydrodynamic trapping system to form four-cell clusters consisting of one pancreatic β-cell and three breast cancer cells. Single-cell traps were typically occupied within seconds of the first cells entering the chip, while loose cells were recovered at the cell outlet.^95^ This technique can especially be useful for modeling microtissue niches and enables tailored cell assemblies. Because of the difficulty of obtaining and maintaining CTC–neutrophil clusters *ex vivo*, the formation of CTC–neutrophil clusters *in vitro* could be a significant step for understanding the metastatic mechanisms of heterotypic clusters. Park *et al.*^96^ employed an inertial-force-assisted droplet microfluidic chip with double spiral channels to recapitulate CTC–neutrophil clusters. The deterministic encapsulation of cells facilitated the pairing of neutrophils and cancer cells with varying ratios. The encapsulated cells spontaneously formed clusters and showed well-defined molecular signatures of CTC–neutrophil clusters.^96^

Antibody-coated devices are also used for the formation of clusters.^97–99^ For example, Chen *et al.*^97^ created a membrane mimetic microfluidic device with antibody-conjugated supported lipid bilayer smart coating to capture viable CTCs and clusters directly from whole blood and demonstrated *ex vivo* culture over weeks. The device was able to promote dynamic clustering of lipid-tethered antibodies to CTC antigens and minimize nonspecific blood cells retention. In another study, capture and culture of PC3 prostate cancer cells were presented by Bichsel *et al.*,^98^ where they expanded clonal PC3 cells on functionalized microwells. They then injected hydrogel matrix formulation into the wells. The hydrogel was degradable by matrix metalloproteinases (MMPs), which is important for 3D cell invasion during metastasis. The hydrogel was used to mimic the extracellular matrix (ECM) surrounding the tumor and to facilitate cell proliferation. The cells were then assessed by microscopy over one week in culture.^98^

Microfluidic formation of cell clusters is also feasible without requiring the functionalization or ECM coating of surface areas. Ong *et al.*^100^ developed a gel-free microfluidic trapping method for 3D culturing of A549 and C3A cell lines and primary bone marrow mesenchymal stem cells [Fig. 4(c)]. This simple device was successful in the formation and immobilization of cell aggregates lodged between microposts in a straight channel.^100^ King *et al.*^101^ created microstructures and cultured BT20, MCF-7, and MCF10A aggregates, mimicking tumor heterogeneity by combining all cell lines in a 1:1:1 ratio. The cell cultures yielded a seeding density of 1–15 cells per microstructure in which the seeding density was dependent on incubation time during seeding [Fig. 4(d)]. They also achieved one to five cell clusters per microstructure demonstrating physical similarity of clinically obtained CTCs and CTC aggregates.^101^

The co-culturing approach has also been done to investigate lymph node micrometastasis with cancer cells and natural killer (NK) cells.^102^ Chandrasekaran *et al.*^102^ created a cell culture device to investigate the efficacy of NK-mediated therapy for targeting lymph node micrometastasis. They fabricated spherical cavities using deep reactive ion etched (DRIE) silicon wafers to form the structures in polydimethylsiloxane (PDMS) by gas expansion molding [Fig. 4(e)].^102^ This technique generated rounded microbubble structures, mimicking deep cortical units of a lymph node and creating clusters about 80–100 *μ*m in diameter.^102^ Choi *et al.*^103^ described an early-stage breast cancer chip model that enabled co-culture of breast tumor spheroids with human mammary ductal epithelial cells and mammary fibroblasts in a compartmentalized 3D structure. This device can be used to evaluate the efficacy and toxicity of an anticancer drug.^103^ Hsiao *et al.*^104^ used a two-layer microfluidic device to co-culture prostate cancer cells, osteoblasts, and endothelial cells from 3D cancer tumors. This method ensures uniform incorporation of all co-culture cell types into each spheroid and keeps the spheroids stationary for easy tracking.^104^

These advancements in microfluidic systems can provide larger quantities of rare cells and clusters *in vitro* and, therefore, offer great promise in expanding cancer metastasis studies. Because formation of such clusters with microfluidics allows controllable cell grouping, it is then possible to create heterotypic CTC clusters, making microfluidics more advantageous among other conventional methods. Successful *ex vivo* expansion of CTC clusters will also enable drug screening and personalized medicine applications. The main challenge now for microfluidic systems is to maintain cell viability, which is usually of concern with patient-derived CTCs.

## V. MICROFLUIDIC SYSTEMS FOR CHARACTERIZATION AND ANALYSIS OF CTCs

Molecular analyses of CTC clusters can provide a deeper understanding of the metastatic process and are, therefore, conducted after isolation or formation of clusters. These molecular techniques include immunostaining, FISH, and real time quantitative polymerase chain reaction (RT-qPCR) (Table III). Single-cell analysis, such as next-generation sequencing (NGS) and mass cytometry technologies, may be done to characterize the genome, transcriptome, methylome, and proteome of tumor cells. RNA sequencing analysis was demonstrated on the CTC clusters captured with the Cluster Chip developed by the Toner group,^51^ suggesting some heterogenous characteristics of CTC clusters and association with other immune cells.^51^ Donato *et al.*^35^ evaluated proteins that mediate hypoxia-driven clustering in *in vivo* mouse models. Single-cell technologies have uncovered insight to the genetic makeup of CTCs over the past few years, but limited cell numbers limit molecular analysis, especially if patient stratification is required.^86^ In addition, challenges in single-cell sequencing include strong stochastic variation and high error rates derived from DNA amplification due to limited quantities of DNAs and mRNAs extracted from CTCs.^86^ Nevertheless, some groups have already started to integrate the use of microfluidics in the downstream analysis of CTCs and CTC clusters. In this section, we describe existing technologies for the analysis of CTCs and CTC clusters.

**TABLE III.** Parameters for characterization of CTCs and CTC clusters in microfluidics.

Microfluidic methods of CTC cluster analysis	Relevant parameters
Cell–cell adhesion assays^98^	Cell–cell adhesion forces
Co-culture^101–103,125,127,142^	Cell–cell interactions
Shear flow experiments^19,101,115^	Velocity lateral displacement within channels
High-resolution imaging^19,95,124^ and immunofluorescence^19,74,92^	Cell viability, size and morphology, extravasation rate, protein expression, and molecular characteristics
RNA isolation and protein isolation,^19^ qRT-PCR,^110^ DNA sequencing,^109^ mass spectrometry,^35^ fluorescence *in situ* hybridization (FISH),^3,93^ and surface protein expression^143^	Gene expression, protein expression, molecular characteristics, and proteomic profile

Mouse models of metastasis offer a great platform for CTC and clusters research and have been combined with microfluidic techniques for further CTC characterization. Williams *et al.*^105^ developed diffuse *in vivo* flow cytometry (DiFC), an optical instrument that allows continuous, noninvasive counting green fluorescent protein expressing CTCs in large blood vessels in mice. They used DiFC to study short-term changes in CTC numbers in multiple myeloma and Lewis lung carcinoma xenograft models.^105^ In an integrated microfluidic device capable of capture and isolation, Armbrecht *et al.*^21^ conducted protein quantification secreted by CTCs. The device captures functionalized magnetic beads and single CTCs and CTC-WBC clusters in individual chambers for the assessment of protein secretion.^21^ A simple, label-free acoustofluidic device was developed by Bai *et al.*^36^ from whole blood samples of mice implanted with 4T1 cells derived from BALB/c mice that expressed the firefly luciferase gene.^36^ This study aimed to create a postoperative evaluation system based on the long-term dynamic detection of CTCs to help in guiding treatment in metastatic cancers.^36^

**FIG. 5.**
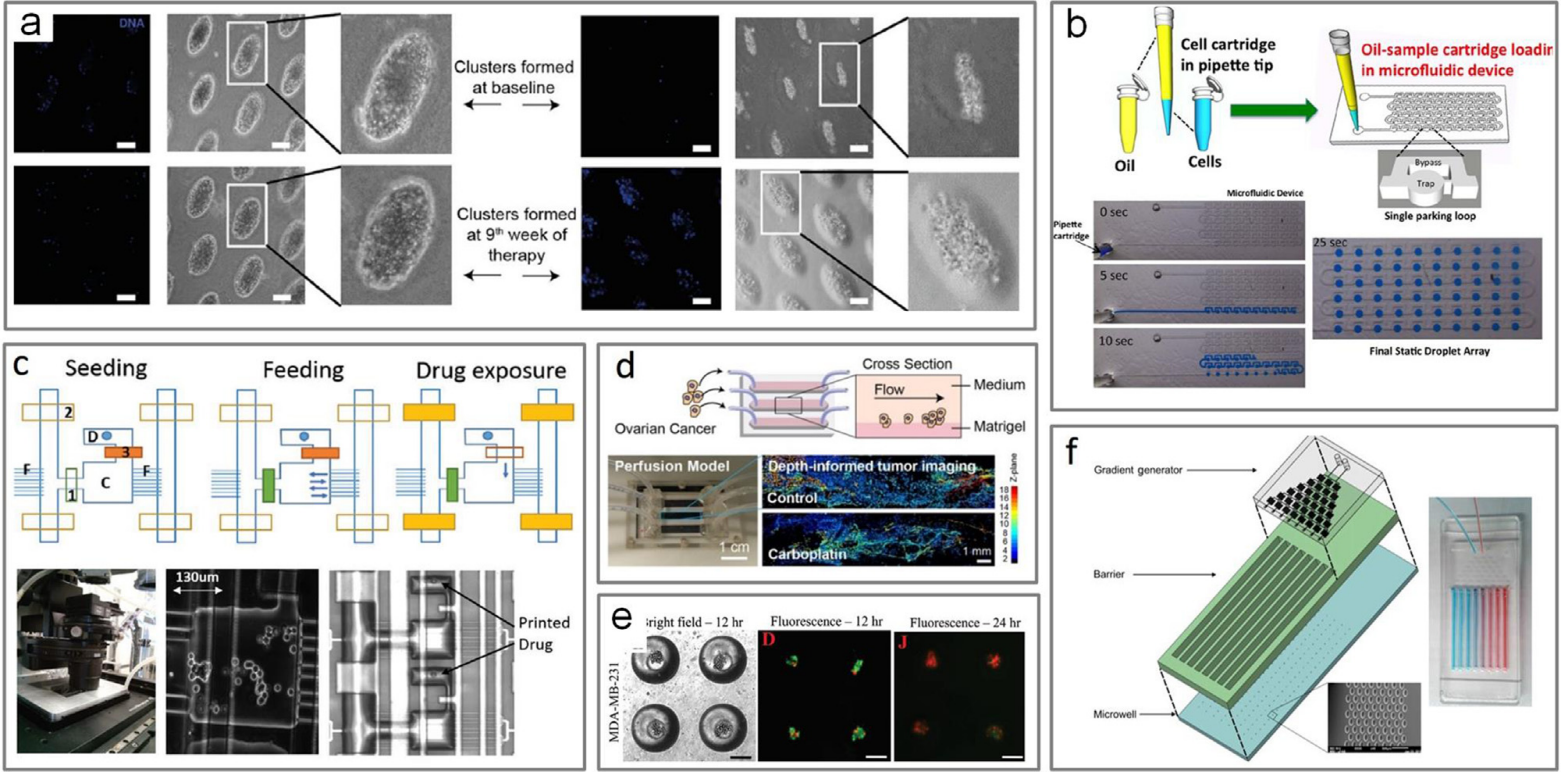
Drug screening and evaluation of targeted therapies in microfluidic devices. (a) Cluster formation in cancer blood samples at baseline and after therapy in a lung cancer patient (left) and breast cancer patient (right). Reproduced with permission from Balakrishnan *et al.*, Sci. Rep. **9**, 1 (2019). Copyright 2019 Authors, licensed under a Creative Commons Attribution (CC BY) license.^92^ (b) Microfluidic drug resistivity assay showing preparation of cartridge by sequential aspiration of oil and cell-laden sample, followed by a single step dispensing into the microfluidic device. Reproduced with permission from Bithi and Vanapalli, Sci. Rep. **7**, 1 (2017). Copyright 2017 Authors, licensed under a Creative Commons Attribution (CC BY) license.^107^ (c) Cell-culture microfluidic device and the drug chambers with printed drug inside. Republished with permission from Desyatnik *et al.*, Adv. Biosyst. **3**, 11 (2019). Copyright John Wiley and Sons Clearance Center, Inc.^94^ (d) Schematic of a perfusion model used to study the impact of fluid flow on treatment resistance and molecular features of 3D ovarian cancer nodules. Reproduced with permission from Khoo *et al.*, Br. J. Cancer **120**, 407 (2019). Copyright 2019 Authors, licensed under a Creative Commons Attribution (CC BY) license.^117^ (e) Cells seeded in microbubbles for evaluation of NK cell mediated therapeutic intervention. Republished with permission from Chandrasekaran *et al.*, Lab Chip **14**, 1 (2019). Copyright Royal Society of Chemistry and Clearance Center, Inc.^102^ (f) Three-dimensional layout of drug assay displaying the layers for the gradient generator, barrier, and microwells. Reproduced with permission from Khoo *et al.*, Sci. Adv. **2**, 7 (2016). Copyright 2016 Authors, Creative Commons Attribution (CC BY) license.^93^

In drug screening, throughput is greatly enhanced in microfluidics owing to the capacity for large numbers of compartments and parallelization. Balakrishnan *et al.*^92^ tested longitudinal treatment response of CTC clusters from patient samples of breast and lung cancer in microwells molded in agar [Fig. 5(a)]. They derived CTCs from cancer patients expanded without prior enrichment and maintained the culture under hypoxic conditions. Drug screening of dissociated solid tissue biopsies has been demonstrated by Eduati *et al.*,^106^ where they used Braille valves to perform combinatorial drug screening. For organoid screening, Au *et al.*^17^ applied a microfluidic platform for culturing hepatic organoids generating arrays of individual, free-floating, 3D hydrogel-based microtissues.^17^ Tu *et al.*^91^ examined migratory behavior of A549 cell aggregates after a screen of drugs in 2D and 3D conditions showing differences in migration patterns between different dimensionalities. In addition, in studying cytotoxic drug resistance, A549 cell aggregates showed a 10–100-fold change in resistance compared to that of a monoculture.^91^ These results suggest that it is important to consider assay types and how they may affect the results of drug screening applications. Ability for increased microenvironmental control and careful geometric and size design of aggregated cells make microfluidics especially conducive for cellular experiments.

In treatment evaluation assays, microchambers can show individual cell/cluster responses to various concentrations of drugs and enable the continuous flow of media for short or long term cell culture. Bithi and Vanapili^107^ created cluster chambers to test chemotherapy drug doxorubicin on MCF-7 cells revealing that cells within a cluster have higher viability than their single-cell counterparts when exposed to the drug [Fig. 5(b)].^107^ Desyatnik *et al.*^94^ employed pneumatic microvalves integrated with cell culturing (up to 7 days) and parallel drug screening to test the chemosensitivity and resistance of MCF-7 and 293T cells [Fig. 5(c)]. Cells were seeded and cultured on the device, then as the valve blocking the drug chamber is opened, drugs flooded and diffused out into the cell chambers.^94^ They also tested the sensitivity of MCF-7 cells to four different drugs at five different doses, each repeated in ten separate chambers.^94^

Genetic and epigenetic characterizations have also been demonstrated via DNA methylation assays of liquid biopsy in a digital chip^108^ as well as single-cell DNA sequencing using droplet microfluidics.^109^ Sun *et al.*^110^ explored the spatial heterogeneity of CTCs within the circulatory system. They used Fluidigm single-cell qRT-PCR to investigate the dynamic expression of EMT-related genes in CTCs during hematogeneous dissemination. They also studied the effects of high shear stress in blood vessels might induce the EMT phenotype in CTCs.^110^ Other organ-specific factors, such as interstitial pressure, oxygen gradient, and stromal cells, could also impact cell phenotype and tumor responses. These, therefore, may be implemented in microfluidic devices to accurately depict *in vivo* CTC clusters. For instance, abnormal fluid pressure also exists in the tumor as elevated interstitial fluid pressure. Shang *et al.*^111^ investigated the interstitial pressure effect on the anticancer drug resistance in CTCs in a pressurized *in vitro* culture device for anticancer drug screening. They found that doxorubicin resistance can be increased by up to 2.5 times under 30 mm Hg due to the overexpression of an efflux transporter gene in human breast cancer cell lines.^111^

Other characterization assays include assays to determine the adhesion strength of CTCs and, therefore, their metastatic potential. Mutlu *et al.*^112^ reported an oscillatory inertial microfluidic system to investigate the cell–cell adhesion strength. They used a repeating fluidic force profile on suspended cell doublets without any biophysical modifications to the cell surface or physiological morphology. They analyzed doublets from a patient-derived breast cancer CTC line.^112^ Another example uses a rectangular channel that allows high shear stresses to be generated under laminar conditions to quantify the relationship between morphological characteristics and adhesion strength for well-spread cells.^113^ Long constriction channels can also be used for cells to transit in, and based on the friction coefficient, the cell adhesion strength may be obtained.^114^

Microfluidic systems have also been used to mimic 3D structural organization and dynamic microenvironment of CTCs for chemoresistance assays. Nath *et al.*^115^ built a perfusion model previously used to assess the effect of flow-induced shear stress on the genetic, molecular, and morphologic features of ovarian cancer in 3D culture over 7 days was modified to evaluate response to carboplatin treatment and photoimmunotherapy [Fig. 5(d)]. They showed the impact of flow-induced shear stress on resistance to carboplatin and modulation of EGFR (epidermal growth factor receptor) -mediated survival pathways in adherent 3D ovarian tumors.^115^ In another example, Chandrasekaran *et al.*^102^ showed the ability to culture tumor cells from surgical explants and studied their sensitivity to a therapeutic approach to target lymph node micrometastases.^102^ They developed spherical cavity culture systems that resemble anatomy of a deep cortical unit of a lymph to determine the effectiveness of liposome-based drug carrier therapies using NK cells conjugated with liposomes [Fig. 5(e)]. Wang *et al.*^116^ created a micropatterned tumor array, which described enabled detailed and dynamic characterization of CAR T cell trafficking toward tumor cell islands and targeting of tumor cells. Their assay allowed for the assessment of CAR T trafficking for immune-oncology research and preclinical assessments of cell-based immunotherapies.^116^ Khoo *et al.*^93^ evaluated drug response of patient-derived CTCs of breast cancer patients throughout the time of treatment using their microwell system [Fig. 5(f)]. Their device was a multi-layer system that includes a channel barrier to prevent fluids with different concentrations from mixing at the cell culture region.^93^ The presence of cancer cells was validated with FISH to identify cells with increased expression of breast cancer-associated markers, TOP2A and CCND1.^93^ In a separate study, the same group also demonstrated a low-dose anti-inflammatory combinatorial treatment of doxorubicin and aspirin using the same device.^117^

With the success of single-cell analysis through microfluidics, the challenge now is to expand these applications to the study of rare cells and clusters. CTC clusters are of interest in the field due to their clinical significance. Downstream analysis of such clusters can provide insight into their genetic makeup, drug response, and resistance mechanisms. It is then important to keep patient-derived cells viable *ex vivo* for as long as possible to conduct such studies. With these in mind, it would be advantageous to integrate isolation systems with cell culture and analysis systems to speed up the post-processing and minimize manual handling.

## VI. MICROFLUIDIC *IN VITRO* MODELS OF CTC CLUSTERS AND METASTASIS

Mechanical cues that are present in circulation such as flow, shear stress,^118^ and hydrostatic pressure^111^ can impact CTC phenotype. High shear forces exerted on CTCs can lead to cell fragmentation and death,^119^ while intermediate shear forces promote extravasation.^46^ Cancer cells in circulation may also have the potential to exploit these mechanical forces for their survival and successful seeding.^47^ It has been shown that hypoxic conditions need to be sustained for primary CTCs to form clusters.^93,120^ In addition, the CTC microenvironment comprises unique features and facilitate interactions between immune cells and CTCs.^34^ Many microfluidic technologies can simulate the metastatic environment permitting accurate physiological and pathological investigations on cancer cell behavior, function, and viability.^121^ While molecular investigations may not be amenable in cell lines, physical and mechanical investigations with microfluidic devices could be possible.

**FIG. 6.**
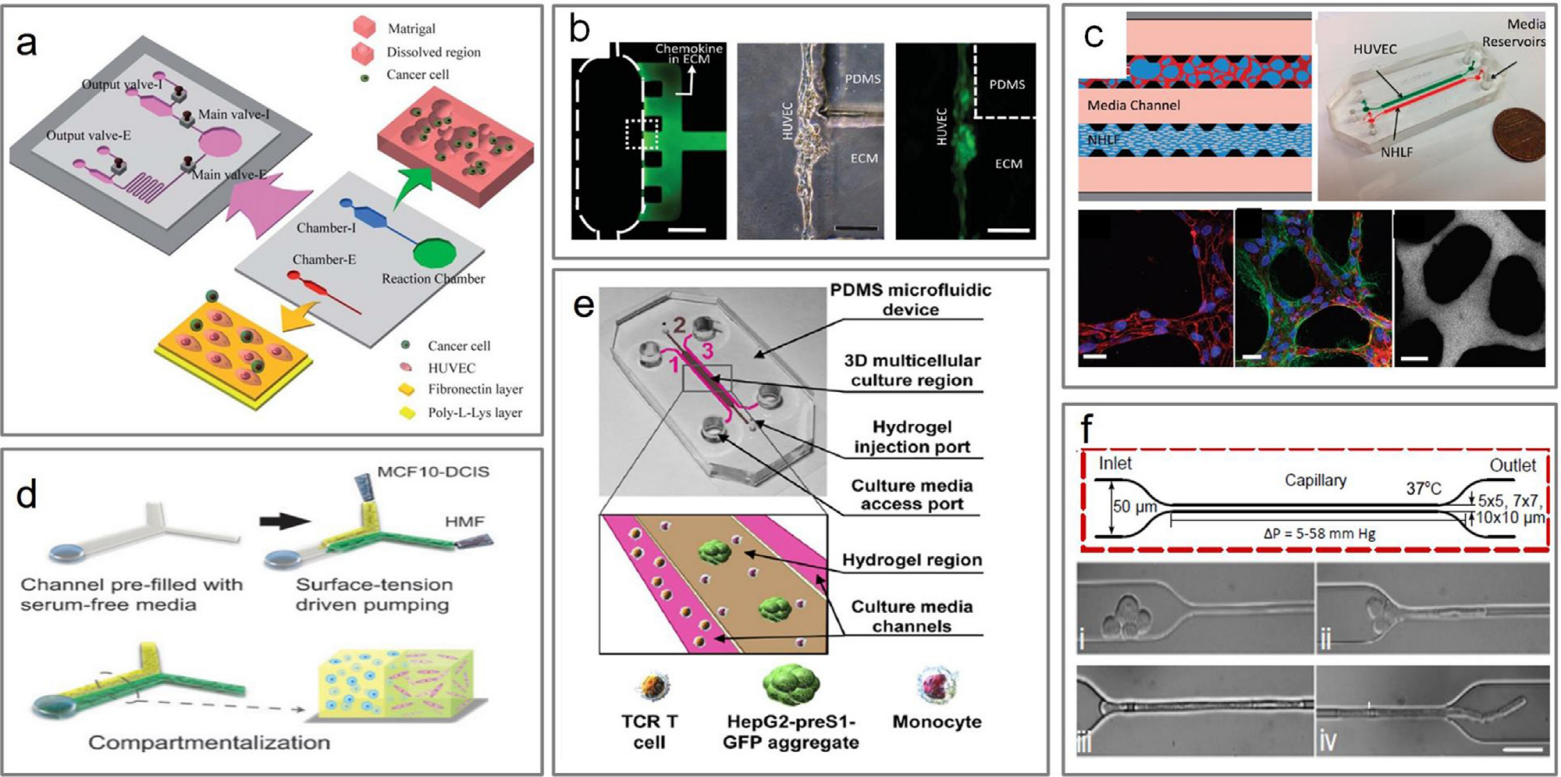
Modeling the metastatic cascade in microfluidics. (a) Integrated cell-based microfluidic chip and the simulation of intra- and extravasation. Republished with permission from Shin *et al.*, Lab Chip **11**, 22 (2011). Copyright Royal Society of Chemistry and Clearance Center, Inc.^122^ (b) Constructed blood vessel model where PDMS pillars between side channels form the scaffold for ECM. Republished with permission from Zhang *et al.*, Lab Chip **12**, 2837 (2012). Copyright Royal Society of Chemistry and Clearance Center, Inc.^123^ (c) A microfluidic microvascular network platform for studying tumor cell extravasation. Reproduced with permission from Chen *et al.*, Integr. Biol. **5**, 1262 (2013).^124^ Copyright 2013 Oxford University Press. (d) 3D compartmentalization and the intravasation of MCF-DCIS cells. Reproduced with permission from Sung *et al.*, Integr. Biol. **3**, 439–450 (2011).^125^ Copyright 2013 Oxford University Press. (e) A microfluidic model of the multicellular tumor microenvironment with a middle hydrogel channel. Reproduced with permission from Lee *et al.*, Front. Immunol. **9**, 416, (2018). Copyright 2018 Authors, licensed under a Creative Commons Attribution (CC BY) license.^127^ (f) Constriction device for cluster migration. Reproduced with permission from Au *et al.*, Proc. Natl. Acad. Sci. **113**, 18 (2016). Copyright 2016 Authors, licensed under a Creative Commons Attribution (CC BY) license.^128^

*In vitro* studies of invasion and metastasis are especially conducive with microfluidic devices with the compartmentalization of microchambers with the use of valves. For instance, both intravasation and extravasation studies were conducted in an integrated microfluidic chip to study the metastatic cascade.^122^ Shin *et al.*^122^ created a device that consists of an intravasation chamber for a 3D culture of cancer cells using a Matrigel matrix and an extravasation chamber [Fig. 6(a)].^122^ Their device demonstrated the detection of metastasized cancer cells by adhesion molecules expressed by epithelial cells.^122^ In another study, Zhang *et al.*^123^ created a microfluidic device that is representative of the principal components of biological blood vessels, including vessel cavity, endothelium, and perivascular matrix [Fig. 6(b)]. Their goal was to study the transendothelial invasion of salivary gland adenoid cystic carcinoma cell aggregates under chemokine stimulation.^123^ A physiologically relevant model of transendothelial extravasation was also created by Chen *et al.*^124^ using a microfluidic platform that incorporates a self-organized 3D microvascular network [Fig. 6(c)]. The device is coupled with the capability for live tracking of single-cell and cell cluster extravasation events, allowing both tumor and endothelial morphological dynamics to be observed. They found that tumor cell transendothelial migration efficiency was higher for cell clusters compared to single cells.^124^ In another example, Sung *et al.*^125^ modeled the transition of ductal carcinoma *in situ* (DCIS) into invasive ductal carcinoma (IDC) in a compartmentalized co-culture system to facilitate the observation of one cell type independently and of distance-dependent effects [Fig. 6(d)].^125^

Microfluidic systems permit higher complexity compared to other conventional 3D culture systems, which allow for the investigations of key roles that other cells or signaling factors play during cancer progression. McCutcheon *et al.*^126^ used a microfluidic platform to distinguish collective migration of neuroclusters from that of individual cells in response to controlled concentration profiles of stromal-derived growth factor (SDF-1). With the ability to precisely form heterotypic cell clusters, Fatsis-Kavalopoulos *et al.*^95^ conducted paracrine signaling studies in a controlled cell assembly generator. They were able to spatially resolve and analyze paracrine effects of β-cell ATP (adenosine triphosphate) release on Ca^2+^ dynamics in three to four breast cancer cells.^95^ Furthermore, careful design and the addition of different cell types, chemokines, and extracellular matrix (ECM) components can model more complex mechanisms. Lee *et al.*^127^ investigated the immunosuppressive role of monocytes and PD-L1/PD-1 signaling on targeting of hepatitis B virus (HBV)-specific T cell receptor-redirected (TCR) T cells using a 3D static microfluidic model [Fig. 6(e)]. They created a 3D co-culture system of target HepG2 cell aggregates, HBV specific TCR T cells, and monocytes within a microfluidic device, where monocytes were suspended together with target cell aggregates in collagen gel.^127^

One of the main features of microfluidic devices is the microsized channels and chambers that offer flexibility in design. Rizvi *et al.*^19^ compared static and flow conditions on ovarian cancer cells and clusters to evaluate the effects of flow on the growth of 3D ovarian micronodules. They found that compared with nonflow cultures, 3D ovarian cancer nodules grown under flow exhibited morphological features indicative of increased EMT and different expression levels of EGFR, E-cadherin, CDC2, and p27Kip1.^19^ In another instance, Au *et al.*^128^ introduced a microfluidic device that mimicked constrictions of the human capillary using 10 × 10-*μ*m^2^ cross-sectional channels [Fig. 6(f)]. They found that >90% of CTC clusters contain up to 20 cells successfully traversed 5–10 *μ*m constrictions even in whole blood. The CTC clusters effectively reorganized into single-file geometries, which substantially reduced their hydrodynamic resistances.^128^ King *et al.*^101^ explored the hemodynamic force effects on cancer cell cluster transport. They used microrenathane microtubes with an inner diameter of 300 *μ*m functionalized with E-selectin to mimic blood vessels.^101^ They were able to simulate CTC-endothelial cell interactions by quantifying rolling velocity and displacement of the cell line aggregates on E-selectin coated surfaces.

Microfluidics has proven to be very useful in creating *in vitro* models used to study the metastatic cascade due the ability to fully capture key features of the immune microenvironment that occur *in vivo*, especially during the metastatic spread.^129^ In addition, physical forces that CTCs experience during their transit in the blood can be precisely mimicked in microchannels. Microfluidic systems also play a role in downstream cellular investigations and allow for high-resolution imaging. Furthermore, *in vitro* systems allow for the use of human-derived cells exclusively without the confounding effects of animal host cells observed in animal models.^129^

## VII. SUMMARY AND OUTLOOK

As microfluidic devices are gaining popularity for cellular manipulation and analysis, these technologies are now also applied in drug screening and cellular characterization. Microfluidic isolation of cells and clusters, including label-free and immunoselection approaches, based on either passive or active methods, have progressed in efficiency and throughput paving the way for liquid biopsy as a relatively easy and minimally invasive way to obtain primary cells for disease monitoring. Current microfluidic cluster isolation methods can achieve high efficiency (up to >99%)^56^ and viability (>90%).^128^ Compared with passive isolation methods, the active methods exhibit higher efficiency but slightly lower cell viability. Active methods are also more complex in operation and would overall need improvement in viability, cost, and simplicity. The ability to isolate CTC clusters is a significant step toward tumor cell characterization and modeling. However, although microfluidic isolation systems have become more sophisticated over the past few years, the rarity of primary cell clusters restricts the extensive characterization and screening of primary cells and clusters. There must then be an improvement in microfluidic systems in areas, including cell retention, viability, sensitivity, and physiological modeling.

Downstream analysis especially at the single-cell level remains to be a challenge. For instance, enzyme-linked immunosorbent assays (ELISA), PCR, western blots, and mass spectrometry require sufficient number of recovered cells per test since low cell densities may not be enough for accurate molecular analysis. With onset low viability of CTCs, there could be even lesser viable cells available for such analyses.^42^ The cell capture rates of different single-cell RNA-seq methods can be as low as >1% or go up to 62%.^130^ In this realm, sample loss needs to be significantly minimized. Much of the cell loss can be attributed to manual handling of samples such as the transfer from one device to another. There are currently limited options in which small cell numbers can be handled with minimal manual interference. Thus, these issues would need to be addressed perhaps with the development of streamlined microfluidic methods.^50^,^61^ For methods of viability assessment, fluorescence labeling is used to track the viability of cells; however, these signals diminish overtime and so the long-term monitoring of cells may be difficult. Other ways of indicating cell viability would be to determine the metabolic and proliferation rates of cells through MTT (3-(4,5-dimethylthiazol-2-yl)-2,5-diphenyltetrazolium bromide), cell cycle, or DNA synthesis assays. Such assays may need to be incorporated into microfluidic platforms to study CTCs.

On the other hand, with such low cell densities, there is then a need for the improvement on the detection sensitivity of many common molecular analysis methods to achieve true single cell analysis. The varied levels of surface marker expression, for example, can cause labeling methods to be inconsistent. Physical detection or a combination of the label-based and physical methods may, therefore, help mitigate the problem during separation.^1^ At the same time, the expansion of CTCs and CTC clusters can take a long time and, thus, necessitates the development of new methods for rapid expansion of patient-derived tumor cells. Even so, personalized medicine still holds much promise with the microfluidic approach, where processing time could be significantly improved with automation and the integration of upstream methods (i.e., isolation, cell expansion/culture, etc.) with downstream analysis.^131^ Routine screening for longitudinal disease prognosis with microfluidics can provide clinicians with patient-specific information and, therefore, guide early personalized therapeutic interventions as well as monitor disease state. With the development of more droplet microfluidic systems, single cell analysis tremendously advanced through use of nano- to picoliter droplets and barcoding.^109,132,133^

Despite much of the advancements and increased complexity in many microfluidic systems, there are still many challenges affecting performance of these devices in CTC and clusters analysis. For one, a general shortcoming of *in vitro* systems is their inability to fully recreate or replace the *in vivo*. Cell clusters formed *in vitro* may exhibit differences in phenotype and gene expression compared to primary tumors which are highly heterogeneous. The use of cancer cell lines may only provide an alternative and be used as surrogates to complement studies of primary cells.^31^ Moreover, several factors contribute to the metastatic cascade and extravasation and, thus, influence CTC seeding patterns. The growth and metastasis of tumor are not only based on the cancer cell simply but also rely on cancer-associated fibroblasts and immune cells within tumors and other supporting tissue that surround the tumors.

Nevertheless, these shortcomings may be diminished in microfluidic systems that can recapitulate the tumor microenvironment through the introduction of different cell types and the application of mechanical stresses and/or chemical cues. Because of the ability to control biological and physical factors, microfluidic systems have the potential to be more comprehensive and accurate models. Another main limitation in microfluidics is that it may require specialized training in operation as well as fabrication, which is due to the lack of established standards in device development. In order to translate these systems into the clinical setting, operation should become simpler and user-friendly. Drug screening platforms also need to be high-throughput comparable to standard well plates used in commercial screening. Because of the limitations brought on by low cell densities, *in vitro* CTC cluster modeling may be necessary to develop better systems for CTC cluster isolation, culture, and analysis.

Microfluidics can also be used for development of *in vitro* models of CTC clusters due to superior spatial control needed to mimic the cellular microenvironment. With the capability to permit precise control of flow, mechano-transduction pathways involved in metastasis may be investigated more closely to identify the biomechanical cues that may promote extravasation, migration, and eventual seeding of tumor cells.^118^ Gravity-based sedimentation into microwells, cell-specific capture through antibody-coated surfaces, or entrapment in microdroplets in microfluidic devices is often used. These approaches allow them to form clusters on-chip while precisely controlling the cell number and position. The wide size-range of cluster models from less than ten cells to thousand cells is achieved to meet various application requirements in personalized medicine. Microwell size and geometries may be tunable by using laser ablation or 3D printing of microwell molds to achieve precise formation of individual cell clusters, minimize cell loss, and prevent cluster dissociation. Additionally, microfluidics offers advantages of small sample consumption, high throughput, and low cost. These systems also enable the visualization of cells at high resolution due to the short distance from the biological samples in the device to the microscope objective. However, there are limitations in imaging larger-sized cell aggregates when the required working distance becomes too large.

Overall, even with the growing research in the field, much has yet to be learned regarding the prognostic value and potential role of CTC clusters in personalized medicine and drug development. Thus, there are opportunities in the development of *in vitro* models that can fully encompass the intricacies of the metastatic cascade. This may be done through the combination of multiple factors such as biochemical signaling and mechanical stimulation or done through the decoupling of such factors that are usually confounding in *in vivo* models. In addition, future opportunities in device development for CTC cluster studies would be in the integration of multiple procedures such as isolation, culture, and downstream analysis in a single device.^86^ This can hopefully provide ease in the processing of biological samples and ultimately offer insight into drug screening and development of personalized patient cancer treatments.

## Data Availability

Data sharing is not applicable to this article as no new data were created or analyzed in this study.

## References

[1] S. Nagrath *et al.*, “ Isolation of rare circulating tumour cells in cancer patients by microchip technology,” Nature 450, 1235–1239 (2007). 10.1038/nature06385 18097410 PMC3090667

[2] N. Aceto *et al.*, “ Circulating tumor cell clusters are oligoclonal precursors of breast cancer metastasis,” Cell 158, 1110–1122 (2014). 10.1016/j.cell.2014.07.013 25171411 PMC4149753

[3] A. Kulasinghe , J. Zhou , L. Kenny , I. Papautsky , and C. Punyadeera , “ Capture of circulating tumour cell clusters using straight microfluidic chips,” Cancers 11, 89 (2019). 10.3390/cancers11010089 PMC6356955 30646614

[4] S. H. Au *et al.*, “ Microfluidic isolation of circulating tumor cell clusters by size and asymmetry,” Sci. Rep. 7, 2433 (2017). 10.1038/s41598-017-01150-3 28550299 PMC5446400

[5] P. Paterlini-Brechot and N. L. Benali , “ Circulating tumor cells (CTC) detection: Clinical impact and future directions,” Cancer Lett. 253, 180–204 (2007). 10.1016/j.canlet.2006.12.014 17314005

[6] M. Giuliano *et al.*, “ Perspective on circulating tumor cell clusters: Why it takes a village to metastasize,” Cancer Res. 78, 845–852 (2018). 10.1158/0008-5472.CAN-17-2748 29437766

[7] C. Wang *et al.*, “ Longitudinally collected CTCs and CTC-clusters and clinical outcomes of metastatic breast cancer,” Breast Cancer Res. Treat. 161, 83–94 (2017). 10.1007/s10549-016-4026-2 27771841

[8] J.-M. Hou *et al.*, “ Clinical significance and molecular characteristics of circulating tumor cells and circulating tumor microemboli in patients with small-cell lung cancer,” J. Clin. Oncol. 30, 525–532 (2012). 10.1200/JCO.2010.33.3716 22253462

[9] Z. Lin *et al.*, “ Recent advances in microfluidic platforms applied in cancer metastasis: Circulating tumor cells' (CTCs) isolation and tumor‐on‐a‐chip,” Small 16, 1903899 (2020). 10.1002/smll.201903899 31747120

[10] X. Cai , F. Janku , Q. Zhan , and J.-B. Fan , “ Accessing genetic information with liquid biopsies,” Trends Genet. 31, 564–575 (2015). 10.1016/j.tig.2015.06.001 26450339

[11] H. Pei , L. Li , Z. Han , Y. Wang , and B. Tang , “ Recent advances in microfluidic technologies for circulating tumor cells: enrichment, single-cell analysis, and liquid biopsy for clinical applications,” Lab Chip 20, 3854–3875 (2020). 10.1039/D0LC00577K 33107879

[12] G. Mehta , A. Y. Hsiao , M. Ingram , G. D. Luker , and S. Takayama , “ Opportunities and challenges for use of tumor spheroids as models to test drug delivery and efficacy,” J. Controlled Release 164, 192–204 (2012). 10.1016/j.jconrel.2012.04.045 PMC3436947 22613880

[13] K. Moshksayan *et al.*, “ Spheroids-on-a-chip: Recent advances and design considerations in microfluidic platforms for spheroid formation and culture,” Sens. Actuators, B 263, 151–176 (2018). 10.1016/j.snb.2018.01.223

[14] R. Vadivelu , H. Kamble , M. Shiddiky , and N.-T. Nguyen , “ Microfluidic technology for the generation of cell spheroids and their applications,” Micromachines 8, 94 (2017). 10.3390/mi8040094

[15] D. J. Jung *et al.*, “ A one-stop microfluidic-based lung cancer organoid culture platform for testing drug sensitivity,” Lab Chip 19, 2854–2865 (2019). 10.1039/C9LC00496C 31367720

[16] N. Dadgar *et al.*, “ A microfluidic platform for cultivating ovarian cancer spheroids and testing their responses to chemotherapies,” Microsyst. Nanoeng. 6, 93 (2020). 10.1038/s41378-020-00201-6 34567703 PMC8433468

[17] S. H. Au , M. Dean Chamberlain , S. Mahesh , M. V. Sefton , and A. R. Wheeler , “ Hepatic organoids for microfluidic drug screening,” Lab Chip 14, 3290–3299 (2014). 10.1039/C4LC00531G 24984750

[18] W. Tang *et al.*, “ Recent advances in microfluidic cell sorting techniques based on both physical and biochemical principles,” Electrophoresis 40, 930–954 (2019). 10.1002/elps.201800361 30311661

[19] I. Rizvi *et al.*, “ Flow induces epithelial-mesenchymal transition, cellular heterogeneity and biomarker modulation in 3D ovarian cancer nodules,” Proc. Natl. Acad. Sci. 110, E1974–E1983 (2013). 10.1073/pnas.1216989110 23645635 PMC3670329

[20] S. Cha *et al.*, “ Cell stretching measurement utilizing viscoelastic particle focusing,” Anal. Chem. 84, 10471–10477 (2012). 10.1021/ac302763n 23163397

[21] L. Armbrecht *et al.*, “ Quantification of protein secretion from circulating tumor cells in microfluidic chambers,” Adv. Sci. 7, 1903237 (2020). 10.1002/advs.201903237 PMC7284199 32537399

[22] Q. Luan *et al.*, “ Non-small cell lung carcinoma spheroid models in agarose microwells for drug response studies,” Lab Chip 22, 2364 (2022). 10.1039/D2LC00244B 35551303 PMC10319040

[23] M. Yu , S. Stott , M. Toner , S. Maheswaran , and D. A. Haber , “ Circulating tumor cells: Approaches to isolation and characterization,” J. Cell Biol. 192, 373–382 (2011). 10.1083/jcb.201010021 21300848 PMC3101098

[24] P. Rostami *et al.*, “ Novel approaches in cancer management with circulating tumor cell clusters,” J. Sci. Adv. Mater. Devices 4, 1–18 (2019). 10.1016/j.jsamd.2019.01.006

[25] N. Venugopal Menon , S. B. Lim , and C. T. Lim , “ Microfluidics for personalized drug screening of cancer,” Curr. Opin. Pharmacol. 48, 155–161 (2019). 10.1016/j.coph.2019.09.008 31634805

[26] P. P. Praharaj , S. K. Bhutia , S. Nagrath , R. L. Bitting , and G. Deep , “ Circulating tumor cell-derived organoids: Current challenges and promises in medical research and precision medicine,” Biochim. Biophys. Acta BBA 1869, 117–127 (2018). 10.1016/j.bbcan.2017.12.005 PMC6054479 29360544

[27] D. Gao *et al.*, “ Organoid cultures derived from patients with advanced prostate cancer,” Cell 159, 176–187 (2014). 10.1016/j.cell.2014.08.016 25201530 PMC4237931

[28] J. Drost and H. Clevers , “ Organoids in cancer research,” Nat. Rev. Cancer 18, 407–418 (2018). 10.1038/s41568-018-0007-6 29692415

[29] X. Qin and C. J. Tape , “ Deciphering organoids: High-dimensional analysis of biomimetic cultures,” Trends Biotechnol. 39, 774–787 (2021). 10.1016/j.tibtech.2020.10.013 33279281

[30] Y. Fang and R. M. Eglen , “ Three-dimensional cell cultures in drug discovery and development,” SLAS Discov. 22, 456–472 (2017). 10.1177/1087057117696795 28520521 PMC5448717

[31] A. N. May , B. D. Crawford , and A. M. Nedelcu , “ *In vitro* model-systems to understand the biology and clinical significance of circulating tumor cell clusters,” Front. Oncol. 8, 63 (2018). 10.3389/fonc.2018.00063 29594043 PMC5858030

[32] S. Heeke , B. Mograbi , C. Alix-Panabières , and P. Hofman , “ Never travel alone: The crosstalk of circulating tumor cells and the blood microenvironment,” Cells 8, 714 (2019). 10.3390/cells8070714 PMC6678604 31337010

[33] N. Aceto , M. Toner , S. Maheswaran , and D. A. Haber , “ En route to metastasis: Circulating tumor cell clusters and epithelial-to-mesenchymal transition,” Trends Cancer 1, 44–52 (2015). 10.1016/j.trecan.2015.07.006 28741562

[34] B. M. Szczerba *et al.*, “ Neutrophils escort circulating tumour cells to enable cell cycle progression,” Nature 566, 553–557 (2019). 10.1038/s41586-019-0915-y 30728496

[35] C. Donato *et al.*, “ Hypoxia triggers the intravasation of clustered circulating tumor cells,” Cell Rep. 32, 108105 (2020). 10.1016/j.celrep.2020.108105 32905777 PMC7487783

[36] X. Bai *et al.*, “ Postoperative evaluation of tumours based on label-free acoustic separation of circulating tumour cells by microstreaming,” Lab Chip 21, 2721–2729 (2021). 10.1039/D1LC00165E 34165474

[37] M. Yu *et al.*, “ Circulating breast tumor cells exhibit dynamic changes in epithelial and mesenchymal composition,” Science 339, 580–584 (2013). 10.1126/science.1228522 23372014 PMC3760262

[38] M. Labelle , S. Begum , and R. O. Hynes , “ Direct signaling between platelets and cancer cells induces an epithelial-mesenchymal-like transition and promotes metastasis,” Cancer Cell 20, 576–590 (2011). 10.1016/j.ccr.2011.09.009 22094253 PMC3487108

[39] K. Hinohara and K. Polyak , “ Intratumoral heterogeneity: More than just mutations,” Trends Cell Biol. 29, 569–579 (2019). 10.1016/j.tcb.2019.03.003 30987806 PMC6579620

[40] S. Gkountela *et al.*, “ Circulating tumor cell clustering shapes DNA methylation to enable metastasis seeding,” Cell 176, 98–112 (2019). 10.1016/j.cell.2018.11.046 30633912 PMC6363966

[41] I. J. Fidler , “ The pathogenesis of cancer metastasis: The ‘seed and soil’ hypothesis revisited,” Nat. Rev. Cancer 3, 453–458 (2003). 10.1038/nrc1098 12778135

[42] I. J. Fidler , “ The relationship of ernbolic homogeneity, number, size and viability to the incidence of experimental metastasis,” Eur. J. Cancer 9, 223–227 (1973). 10.1016/S0014-2964(73)80022-2 4787857

[43] B. L. Khoo *et al.*, “ Clinical validation of an ultra high-throughput spiral microfluidics for the detection and enrichment of viable circulating tumor cells,” PLoS One 9, e99409 (2014). 10.1371/journal.pone.0099409 24999991 PMC4085042

[44] J. F. Dorsey *et al.*, “ Tracking viable circulating tumor cells (CTCs) in the peripheral blood of non-small cell lung cancer (NSCLC) patients undergoing definitive radiation therapy: Pilot study results: CTCs in RT-treated NSCLC patients,” Cancer 121, 139–149 (2015). 10.1002/cncr.28975 25241991 PMC4270850

[45] M. J. Mitchell *et al.*, “ Lamin A/C deficiency reduces circulating tumor cell resistance to fluid shear stress,” Am. J. Physiol.-Cell Physiol. 309, C736–C746 (2015). 10.1152/ajpcell.00050.2015 26447202 PMC4725441

[46] G. Follain *et al.*, “ Hemodynamic forces tune the arrest, adhesion, and extravasation of circulating tumor cells,” Dev. Cell 45, 33–52 (2018). 10.1016/j.devcel.2018.02.015 29634935

[47] G. Follain *et al.*, “ Fluids and their mechanics in tumour transit: Shaping metastasis,” Nat. Rev. Cancer 20, 107–124 (2020). 10.1038/s41568-019-0221-x 31780785

[48] R. Fan , “ Circulatory shear flow alters the viability and proliferation of circulating colon cancer cells,” Sci. Rep. 6, 27073 (2016). 10.1038/srep27073 27255403 PMC4891768

[49] K. Alvarado-Estrada *et al.*, “ Circulatory shear stress induces molecular changes and side population enrichment in primary tumor-derived lung cancer cells with higher metastatic potential,” Sci. Rep. 11, 2800 (2021). 10.1038/s41598-021-82634-1 33531664 PMC7854722

[50] J. Zhou *et al.*, “ The label-free separation and culture of tumor cells in a microfluidic biochip,” Analyst 145, 1706–1715 (2020). 10.1039/C9AN02092F 31895371

[51] A. F. Sarioglu *et al.*, “ A microfluidic device for label-free, physical capture of circulating tumor cell clusters,” Nat. Methods 12, 685–691 (2015). 10.1038/nmeth.3404 25984697 PMC4490017

[52] L. Zhang *et al.*, “ The identification and characterization of breast cancer CTCs competent for brain metastasis,” Sci. Transl. Med. 5, 180ra48–180ra48 (2013). 10.1126/scitranslmed.3005109 PMC3863909 23576814

[53] X. Qiu , J. De Jesus , M. Pennell , M. Troiani , and J. B. Haun , “ Microfluidic device for mechanical dissociation of cancer cell aggregates into single cells,” Lab Chip 15, 339–350 (2015). 10.1039/C4LC01126K 25377468 PMC4301619

[54] R. Vaidyanathan , R. H. Soon , P. Zhang , K. Jiang , and C. T. Lim , “ Cancer diagnosis: From tumor to liquid biopsy and beyond,” Lab Chip 19, 11–34 (2018). 10.1039/C8LC00684A 30480287

[55] J. Zhou , P. Mukherjee , H. Gao , Q. Luan , and I. Papautsky , “ Label-free microfluidic sorting of microparticles,” APL Bioeng. 3, 041504 (2019). 10.1063/1.5120501 31832577 PMC6906121

[56] X. Xu *et al.*, “ Recent progress of inertial microfluidic-based cell separation,” Analyst 146, 7070–7086 (2021). 10.1039/D1AN01160J 34761757

[57] S.-B. Cheng *et al.*, “ High-efficiency capture of individual and cluster of circulating tumor cells by a microchip embedded with three-dimensional poly(dimethylsiloxane) scaffold,” Anal. Chem. 88, 6773–6780 (2016). 10.1021/acs.analchem.6b01130 27291464

[58] S. L. Stott *et al.*, “ Isolation of circulating tumor cells using a microvortex-generating herringbone-chip,” Proc. Natl. Acad. Sci. 107, 18392–18397 (2010). 10.1073/pnas.1012539107 20930119 PMC2972993

[59] M.-H. Park *et al.*, “ Enhanced isolation and release of circulating tumor cells using nanoparticle binding and ligand exchange in a microfluidic chip,” J. Am. Chem. Soc. 139, 2741–2749 (2017). 10.1021/jacs.6b12236 28133963 PMC5506378

[60] C. W. Shields IV , C. D. Reyes , and G. P. López , “ Microfluidic cell sorting: A review of the advances in the separation of cells from debulking to rare cell isolation,” Lab Chip 15, 1230–1249 (2015). 10.1039/C4LC01246A 25598308 PMC4331226

[61] Q. Luan , C. Macaraniag , J. Zhou , and I. Papautsky , “ Microfluidic systems for hydrodynamic trapping of cells and clusters,” Biomicrofluidics 14, 031502 (2020). 10.1063/5.0002866 34992704 PMC8719525

[62] M. Hosokawa *et al.*, “ Size-based isolation of circulating tumor cells in lung cancer patients using a microcavity array system,” PLoS One 8, e67466 (2013). 10.1371/journal.pone.0067466 23840710 PMC3696066

[63] M. Hayashi *et al.*, “ Size-based detection of sarcoma circulating tumor cells and cell clusters,” Oncotarget 8, 78965–78977 (2017). 10.18632/oncotarget.20697 29108279 PMC5668012

[64] M.-D. Zhou *et al.*, “ Separable bilayer microfiltration device for viable label-free enrichment of circulating tumour cells,” Sci. Rep. 4, 7392 (2014). 10.1038/srep07392 25487434 PMC4260227

[65] H. Cho *et al.*, “ Microfluidic technologies for circulating tumor cell isolation,” Analyst 143, 2936–2970 (2018). 10.1039/C7AN01979C 29796523

[66] L. R. Huang , E. C. Cox , R. H. Austin , and J. C. Sturm , “ Continuous particle separation through deterministic lateral displacement,” Science 304, 987–990 (2004). 10.1126/science.1094567 15143275

[67] J. F. Edd *et al.*, “ Microfluidic concentration and separation of circulating tumor cell clusters from large blood volumes,” Lab Chip 20, 558–567 (2020). 10.1039/C9LC01122F 31934715 PMC7469923

[68] M. Zeinali *et al.*, “ High-throughput label-free isolation of heterogeneous circulating tumor cells and CTC clusters from non-small-cell lung cancer patients,” Cancers 12, 127 (2020). 10.3390/cancers12010127 PMC7016759 31947893

[69] H. W. Hou *et al.*, “ Isolation and retrieval of circulating tumor cells using centrifugal forces,” Sci. Rep. 3, 1259 (2013). 10.1038/srep01259 23405273 PMC3569917

[70] S. C. Hur , A. J. Mach , and D. D. Carlo , “ High-throughput size-based rare cell enrichment using microscale vortices,” Biomicrofluidics 5, 022206 (2011). 10.1063/1.3576780 PMC3171489 21918676

[71] E. Sollier *et al.*, “ Size-selective collection of circulating tumor cells using Vortex technology,” Lab Chip 14, 63–77 (2014). 10.1039/C3LC50689D 24061411

[72] J. Zhou *et al.*, “ Isolation of cells from whole blood using shear-induced diffusion,” Sci. Rep. 8, 9411 (2018). 10.1038/s41598-018-27779-2 29925931 PMC6010421

[73] J. Zhou *et al.*, “ Isolation of circulating tumor cells in non-small-cell-lung-cancer patients using a multi-flow microfluidic channel,” Microsyst. Nanoeng. 5, 8 (2019). 10.1038/s41378-019-0045-6 31057935 PMC6387977

[74] Z. Zhang *et al.*, “ Expansion of CTCs from early stage lung cancer patients using a microfluidic co-culture model,” Oncotarget 5, 12383–12397 (2014). 10.18632/oncotarget.2592 25474037 PMC4323004

[75] X. Jiang *et al.*, “ Microfluidic isolation of platelet-covered circulating tumor cells,” Lab Chip 17, 3498–3503 (2017). 10.1039/C7LC00654C 28932842 PMC5690580

[76] S.-B. Cheng *et al.*, “ Three-dimensional scaffold chip with thermosensitive coating for capture and reversible release of individual and cluster of circulating tumor cells,” Anal. Chem. 89, 7924–7932 (2017). 10.1021/acs.analchem.7b00905 28661138

[77] J. Kitz , D. Goodale , C. Postenka , L. E. Lowes , and A. L. Allan , “ EMT-independent detection of circulating tumor cells in human blood samples and pre-clinical mouse models of metastasis,” Clin. Exp. Metastasis 38, 97–108 (2021). 10.1007/s10585-020-10070-y 33415568 PMC7882592

[78] M. A. Burguillos *et al.*, “ Microchannel acoustophoresis does not impact survival or function of microglia, leukocytes or tumor cells,” PLoS One 8, e64233 (2013). 10.1371/journal.pone.0064233 23724038 PMC3664584

[79] X. Ding *et al.*, “ Cell separation using tilted-angle standing surface acoustic waves,” Proc. Natl. Acad. Sci. 111, 12992–12997 (2014). 10.1073/pnas.1413325111 25157150 PMC4246961

[80] P. Li *et al.*, “ Acoustic separation of circulating tumor cells,” Proc. Natl. Acad. Sci. 112, 6 (2015). 25848039 10.1073/pnas.1504484112 PMC4413297

[81] P. Augustsson , C. Magnusson , M. Nordin , H. Lilja , and T. Laurell , “ Microfluidic, label-free enrichment of prostate cancer cells in blood based on acoustophoresis,” Anal. Chem. 84, 7954 (2012). 10.1021/ac301723s 22897670 PMC3445767

[82] M. Wu *et al.*, “ Circulating tumor cell phenotyping via high‐throughput acoustic separation,” Small 14, 1801131 (2018). 10.1002/smll.201801131 PMC6105522 29968402

[83] T.-K. Chiu *et al.*, “ Optically-induced-dielectrophoresis (ODEP)-based cell manipulation in a microfluidic system for high-purity isolation of integral circulating tumor cell (CTC) clusters based on their size characteristics,” Sens. Actuators, B 258, 1161–1173 (2018). 10.1016/j.snb.2017.12.003

[84] S. Lin *et al.*, “ A flyover style microfluidic chip for highly purified magnetic cell separation,” Biosens. Bioelectron. 129, 175–181 (2019). 10.1016/j.bios.2018.12.058 30710755

[85] L. Q. Do *et al.*, “ Dielectrophoresis microfluidic enrichment platform with built-in capacitive sensor for rare tumor cell detection,” BioChip J. 12, 114–122 (2018). 10.1007/s13206-017-2204-x

[86] F. Castro-Giner and N. Aceto , “ Tracking cancer progression: From circulating tumor cells to metastasis,” Genome Med. 12, 31 (2020). 10.1186/s13073-020-00728-3 32192534 PMC7082968

[87] J. R. Rettig and A. Folch , “ Large-scale single-cell trapping and imaging using microwell arrays,” Anal. Chem. 77, 5628–5634 (2005). 10.1021/ac0505977 16131075

[88] J. M. Kelm , N. E. Timmins , C. J. Brown , M. Fussenegger , and L. K. Nielsen , “ Method for generation of homogeneous multicellular tumor spheroids applicable to a wide variety of cell types,” Biotechnol. Bioeng. 83, 173–180 (2003). 10.1002/bit.10655 12768623

[89] R. K. Vadivelu , H. Kamble , A. Munaz , and N.-T. Nguyen , “ Liquid marble as bioreactor for engineering three-dimensional toroid tissues,” Sci. Rep. 7, 12388 (2017). 10.1038/s41598-017-12636-5 28959016 PMC5620055

[90] D. M. Headen , J. R. García , and A. J. García , “ Parallel droplet microfluidics for high throughput cell encapsulation and synthetic microgel generation,” Microsyst. Nanoeng. 4, 17076 (2018). 10.1038/micronano.2017.76

[91] T.-Y. Tu *et al.*, “ Rapid prototyping of concave microwells for the formation of 3D multicellular cancer aggregates for drug screening,” Adv. Healthcare Mater. 3, 609–616 (2013). 10.1002/adhm.201300151 PMC4038742 23983140

[92] A. Balakrishnan *et al.*, “ Circulating tumor cell cluster phenotype allows monitoring response to treatment and predicts survival,” Sci. Rep. 9, 7933 (2019). 10.1038/s41598-019-44404-y 31138856 PMC6538674

[93] B. L. Khoo *et al.*, “ Liquid biopsy and therapeutic response: Circulating tumor cell cultures for evaluation of anticancer treatment,” Sci. Adv. 2, e1600274 (2016). 10.1126/sciadv.1600274 27453941 PMC4956185

[94] I. Desyatnik *et al.*, “ An integrated microfluidics approach for personalized cancer drug sensitivity and resistance assay,” Adv. Biosyst. 3, 1900001 (2019). 10.1002/adbi.201900001 32648689

[95] N. Fatsis-Kavalopoulos *et al.*, “ Formation of precisely composed cancer cell clusters using a cell assembly generator (CAGE) for studying paracrine signaling at single-cell resolution,” Lab Chip 19, 1071–1081 (2019). 10.1039/C8LC01153B 30783638

[96] J. Park , S. Park , K. A. Hyun , and H.-I. Jung , “ Microfluidic recapitulation of circulating tumor cell–neutrophil clusters via double spiral channel-induced deterministic encapsulation,” Lab Chip 21, 3483–3497 (2021). 10.1039/D1LC00433F 34309611

[97] J.-Y. Chen *et al.*, “ Sensitive and specific biomimetic lipid coated microfluidics to isolate viable circulating tumor cells and microemboli for cancer detection,” PLoS One 11, e0149633 (2016). 10.1371/journal.pone.0149633 26938471 PMC4777486

[98] C. A. Bichsel *et al.*, “ Diagnostic microchip to assay 3D colony-growth potential of captured circulating tumor cells,” Lab Chip 12, 2313 (2012). 10.1039/c2lc40130d 22565166

[99] S. W. Shaner *et al.*, “ Design and production of a novel microfluidic device for the capture and isolation of circulating tumor cell clusters,” AIP Adv. 9, 065313 (2019). 10.1063/1.5084736

[100] S.-M. Ong *et al.*, “ A gel-free 3D microfluidic cell culture system,” Biomaterials 29, 3237–3244 (2008). 10.1016/j.biomaterials.2008.04.022 18455231

[101] M. R. King *et al.*, “ A physical sciences network characterization of circulating tumor cell aggregate transport,” Am. J. Physiol.-Cell Physiol. 308, C792–C802 (2015). 10.1152/ajpcell.00346.2014 25788574 PMC4436994

[102] S. Chandrasekaran , M. J. McGuire , and M. R. King , “ Sweeping lymph node micrometastases off their feet: an engineered model to evaluate natural killer cell mediated therapeutic intervention of circulating tumor cells that disseminate to the lymph nodes,” Lab Chip 14, 118–127 (2014). 10.1039/C3LC50584G 23934067

[103] Y. Choi *et al.*, “ A microengineered pathophysiological model of early-stage breast cancer,” Lab Chip 15, 3350–3357 (2015). 10.1039/C5LC00514K 26158500 PMC4524879

[104] A. Y. Hsiao *et al.*, “ Microfluidic system for formation of PC-3 prostate cancer co-culture spheroids,” Biomaterials 30, 3020–3027 (2009). 10.1016/j.biomaterials.2009.02.047 19304321 PMC2675053

[105] A. L. Williams , J. E. Fitzgerald , F. Ivich , E. D. Sontag , and M. Niedre , “ Short-term circulating tumor cell dynamics in mouse xenograft models and implications for liquid biopsy,” Front. Oncol. 10, 601085 (2020). 10.3389/fonc.2020.601085 33240820 PMC7677561

[106] F. Eduati *et al.*, “ A microfluidics platform for combinatorial drug screening on cancer biopsies,” Nat. Commun. 9, 2434 (2018). 10.1038/s41467-018-04919-w 29934552 PMC6015045

[107] S. S. Bithi and S. A. Vanapalli , “ Microfluidic cell isolation technology for drug testing of single tumor cells and their clusters,” Sci. Rep. 7, 41707 (2017). 10.1038/srep41707 28150812 PMC5288702

[108] C. M. O'Keefe *et al.*, “ Facile profiling of molecular heterogeneity by microfluidic digital melt,” Sci. Adv. 4, aat6459 (2018). 10.1126/sciadv.aat6459 PMC6157960 30263958

[109] M. Pellegrino *et al.*, “ High-throughput single-cell DNA sequencing of acute myeloid leukemia tumors with droplet microfluidics,” Genome Res. 28, 1345–1352 (2018). 10.1101/gr.232272.117 30087104 PMC6120635

[110] Y.-F. Sun *et al.*, “ Circulating tumor cells from different vascular sites exhibit spatial heterogeneity in epithelial and mesenchymal composition and distinct clinical significance in hepatocellular carcinoma,” Clin. Cancer Res. 24, 547–559 (2018). 10.1158/1078-0432.CCR-17-1063 29070526

[111] M. Shang *et al.*, “ Microfluidic studies of hydrostatic pressure-enhanced doxorubicin resistance in human breast cancer cells,” Lab Chip 21, 746–754 (2021). 10.1039/D0LC01103G 33502419

[112] B. R. Mutlu *et al.*, “ In-flow measurement of cell–cell adhesion using oscillatory inertial microfluidics,” Lab Chip 20, 1612–1620 (2020). 10.1039/D0LC00089B 32301448 PMC7495683

[113] K. V. Christ , K. B. Williamson , K. S. Masters , and K. T. Turner , “ Measurement of single-cell adhesion strength using a microfluidic assay,” Biomed. Microdevices 12, 443–455 (2010). 10.1007/s10544-010-9401-x 20213215

[114] M. Wei , F. Zhang , R. Zhang , J.-M. Lin , and N. Yang , “ High-throughput characterization of cell adhesion strength using long-channel constriction-based microfluidics,” ACS Sens. 6, 2838–2844 (2021). 10.1021/acssensors.1c01037 34279900

[115] S. Nath *et al.*, “ Flow-induced shear stress confers resistance to carboplatin in an adherent three-dimensional model for ovarian cancer: A role for EGFR-targeted photoimmunotherapy informed by physical stress,” J. Clin. Med. 9, 924 (2020). 10.3390/jcm9040924 PMC7230263 32231055

[116] X. Wang *et al.*, “ Dynamic profiling of antitumor activity of CAR T cells using micropatterned tumor arrays,” Adv. Sci. 6, 1901829 (2019). 10.1002/advs.201901829 PMC6891905 31832320

[117] B. L. Khoo *et al.*, “ Low-dose anti-inflammatory combinatorial therapy reduced cancer stem cell formation in patient-derived preclinical models for tumour relapse prevention,” Br. J. Cancer 120, 407–423 (2019). 10.1038/s41416-018-0301-9 30713340 PMC6461953

[118] C. M. Novak , E. N. Horst , C. C. Taylor , C. Z. Liu , and G. Mehta , “ Fluid shear stress stimulates breast cancer cells to display invasive and chemoresistant phenotypes while upregulating PLAU in a 3D bioreactor,” Biotechnol. Bioeng. 116, 3084–3097 (2019). 10.1002/bit.27119 31317530 PMC6774895

[119] M. B. Headley *et al.*, “ Visualization of immediate immune responses to pioneer metastatic cells in the lung,” Nature 531, 513–517 (2016). 10.1038/nature16985 26982733 PMC4892380

[120] B. L. Khoo *et al.*, “ Short-term expansion of breast circulating cancer cells predicts response to anti-cancer therapy,” Oncotarget 6, 15578–15593 (2015). 10.18632/oncotarget.3903 26008969 PMC4558172

[121] H. Chen *et al.*, “ Microfluidic models of physiological or pathological flow shear stress for cell biology, disease modeling and drug development,” TrAC Trends Anal. Chem. 117, 186–199 (2019). 10.1016/j.trac.2019.06.023

[122] M. K. Shin , S. K. Kim , and H. Jung , “ Integration of intra- and extravasation in one cell-based microfluidic chip for the study of cancer metastasis,” Lab Chip 11, 3880 (2011). 10.1039/c1lc20671k 21975823

[123] Q. Zhang , T. Liu , and J. Qin , “ A microfluidic-based device for study of transendothelial invasion of tumor aggregates in realtime,” Lab Chip 12, 2837 (2012). 10.1039/c2lc00030j 22648473

[124] M. B. Chen , J. A. Whisler , J. S. Jeon , and R. D. Kamm , “ Mechanisms of tumor cell extravasation in an in vitro microvascular network platform,” Integr. Biol. 5, 1262 (2013). 10.1039/c3ib40149a PMC4038741 23995847

[125] K. E. Sung *et al.*, “ Transition to invasion in breast cancer: a microfluidic *in vitro* model enables examination of spatial and temporal effects,” Integr. Biol. 3, 439–450 (2011). 10.1039/C0IB00063A PMC3094750 21135965

[126] S. McCutcheon *et al.*, “ *In vitro* formation of neuroclusters in microfluidic devices and cell migration as a function of stromal-derived growth factor 1 gradients,” Cell Adhes. Migr. 11, 1–12 (2017). 10.1080/19336918.2015.1131388 PMC5308225 26744909

[127] S. W. L. Lee *et al.*, “ Characterizing the role of monocytes in T cell cancer immunotherapy using a 3D microfluidic model,” Front. Immunol. 9, 416 (2018). 10.3389/fimmu.2018.00416 29559973 PMC5845585

[128] S. H. Au *et al.*, “ Clusters of circulating tumor cells traverse capillary-sized vessels,” Proc. Natl. Acad. Sci. 113, 4947–4952 (2016). 10.1073/pnas.1524448113 27091969 PMC4983862

[129] A. Boussommier-Calleja , R. Li , M. B. Chen , S. C. Wong , and R. D. Kamm , “ Microfluidics: A new tool for modeling cancer–immune interactions,” Trends Cancer 2, 6–19 (2016). 10.1016/j.trecan.2015.12.003 26858990 PMC4743529

[130] T. M. Yamawaki *et al.*, “ Systematic comparison of high-throughput single-cell RNA-seq methods for immune cell profiling,” BMC Genomics 22, 66 (2021). 10.1186/s12864-020-07358-4 33472597 PMC7818754

[131] L. Mathur , M. Ballinger , R. Utharala , and C. A. Merten , “ Microfluidics as an enabling technology for personalized cancer therapy,” Small 16, 1904321 (2020). 10.1002/smll.201904321 31747127

[132] A. Kulesa , J. Kehe , J. E. Hurtado , P. Tawde , and P. C. Blainey , “ Combinatorial drug discovery in nanoliter droplets,” Proc. Natl. Acad. Sci. 115, 6685–6690 (2018). 10.1073/pnas.1802233115 29899149 PMC6042083

[133] D. Pekin *et al.*, “ Quantitative and sensitive detection of rare mutations using droplet-based microfluidics,” Lab Chip 11, 2156 (2011). 10.1039/c1lc20128j 21594292

[134] T. Takebe and J. M. Wells , “ Organoids by design,” Science 364, 956–959 (2019). 10.1126/science.aaw7567 31171692 PMC8212787

[135] S. Sharma *et al.*, “ Circulating tumor cell isolation, culture, and downstream molecular analysis,” Biotechnol. Adv. 36, 1063–1078 (2018). 10.1016/j.biotechadv.2018.03.007 29559380 PMC5971144

[136] J. M. Ayuso , K.-Y. Park , M. Virumbrales-Muñoz , and D. J. Beebe , “ Toward improved in vitro models of human cancer,” APL Bioeng. 5, 010902 (2021). 10.1063/5.0026857 33532672 PMC7822630

[137] F. Duzagac , G. Saorin , L. Memeo , V. Canzonieri , and F. Rizzolio , “ Microfluidic organoids-on-a-chip: Quantum leap in cancer research,” Cancers 13, 737 (2021). 10.3390/cancers13040737 33578886 PMC7916612

[138] S. K. Kim , Y. H. Kim , S. Park , and S.-W. Cho , “ Organoid engineering with microfluidics and biomaterials for liver, lung disease, and cancer modeling,” Acta Biomater. 132, 37–51 (2021). 10.1016/j.actbio.2021.03.002 33711526

[139] V. Velasco , S. A. Shariati , and R. Esfandyarpour , “ Microtechnology-based methods for organoid models,” Microsyst. Nanoeng. 6, 76 (2020). 10.1038/s41378-020-00185-3 34567686 PMC8433138

[140] M. B. Chen *et al.*, “ On-chip human microvasculature assay for visualization and quantification of tumor cell extravasation dynamics,” Nat. Protoc. 12, 865–880 (2017). 10.1038/nprot.2017.018 28358393 PMC5509465

[141] J. W. Kamande *et al.*, “ Modular microsystem for the isolation, enumeration, and phenotyping of circulating tumor cells in patients with pancreatic cancer,” Anal. Chem. 85, 9092–9100 (2013). 10.1021/ac401720k 23947293 PMC3832346

[142] B. L. Khoo *et al.*, “ Expansion of patient-derived circulating tumor cells from liquid biopsies using a CTC microfluidic culture device,” Nat. Protoc. 13, 34–58 (2018). 10.1038/nprot.2017.125 29215634

[143] P. M. Aldridge *et al.*, “ Prismatic deflection of live tumor cells and cell clusters,” ACS Nano 12, 12692–12700 (2018). 10.1021/acsnano.8b07616 30444600 PMC6472972

